# Taxonomic reinvestigation of the genus *Tetradesmus* (Scenedesmaceae; Sphaeropleales) based on morphological characteristics and chloroplast genomes

**DOI:** 10.3389/fpls.2024.1303175

**Published:** 2024-02-14

**Authors:** Hyeon Shik Cho, JunMo Lee

**Affiliations:** ^1^ Department of Oceanography, Kyungpook National University, Daegu, Republic of Korea; ^2^ Kyungpook Institute of Oceanography, Kyungpook National University, Daegu, Republic of Korea

**Keywords:** *Tetradesmus*, cellular arrangement, coenobial type, chloroplast genome, phylogeny

## Abstract

The genus *Tetradesmus* (Scenedesmaceae; Sphaeropleales) comprises one of the most abundant green algae in freshwater environments. It includes morphologically diverse species that exhibit bundle-like, plane-arranged coenobia, and unicells, because several different *Scenedesmus*-like groups were integrated into this genus based on phylogenetic analysis. Nevertheless, there is no clear information regarding the phylogenetic relationship of *Tetradesmus* species, determined using several marker genes, because of low phylogenetic support and insufficient molecular data. Currently, genome information is available from diverse taxa, which could provide high-resolution evolutionary relationships. In particular, phylogenetic studies using chloroplast genomes demonstrated the potential to establish high-resolution phylogenetic relationships. However, only three chloroplast genomes are available from the genus *Tetradesmus*. In this study, we newly generated 9 chloroplast genomes from *Tetradesmus* and constructed a high-resolution phylogeny using a concatenated alignment of 69 chloroplast protein sequences. We also report one novel species (*T*. *lancea*), one novel variety (*T*. *obliquus* var. *spiraformis*), and two novel formae (*T*. *dissociatus* f. *oviformis*, *T*. *obliquus* f. *rectilineare*) within the genus *Tetradesmus* based on morphological characteristics (e.g., cellular arrangements and coenobial types) and genomic features (e.g., different exon–intron structures in chloroplast genomes). Moreover, we taxonomically reinvestigated the genus *Tetradesmus* based on these results. Altogether, our study can provide a comprehensive understanding of the taxonomic approaches for investigating this genus.

## 1 Introduction

The genus *Tetradesmus* G.M.Smith (Scenedesmaceae; Sphaeropleales) comprises a ubiquitous group of unicellular green algae found in aquatic and terrestrial environments, including rocks, sand, and soil (Tsarenko and John, 2011). This genus was characterized by spindle-shaped cells that generally form a bundle-like coenobium (Smith, 1913). Phylogenetic analyses have led to the integration of several *Scenedesmus*-like species, including *Tetradesmus wisconsinensis* G.M.Smith as the type species, into the genus *Acutodesmus* (Hegewald) Tsarenko. Formerly a subgenus within the genus *Scenedesmus* Myen, *Acutodesmus* now encompasses not only coenobia with a bundle-like structure but also those arranged on a plane, along with unicells, showing diverse morphological characteristics (Tsarenko and Petlevanny, 2001; Hegewald et al., 2013). However, ten *Acutodesmus* taxa have once again been relocated to the genus *Tetradesmus* because the genus *Acutodesmus* established in 2001, incorporating the type species *A*. *wisconsinensis*, but this species was initially described as the type species for the earlier genus *Tetradesmus* by G.M.Smith in 1913 (Wynne and Hallan, 2015; Wynne and Guiry, 2016; Lewis and Flechtner, 2019). Because of the complicated taxonomic history of the genus *Tetradesmus*, synonyms of *Tetradesmus* species are still used, especially in biotechnological research [e.g., *Scenedesmus dimorphus* (Turpin) Kützing and *Acutodesmus obliquus* (Turpin) Hegewald & Hanagata; Talarek-Karwel et al., 2020; Hu et al., 2021].

The phylogenetic relationships of *Tetradesmus* species have been extensively investigated using 18S ribosomal RNA or ITS2 sequences (Hegewald and Hanagata, 2000; Eliaš et al., 2010; Hegewald et al., 2010; Hegewald et al., 2013) and chloroplast genes (e.g., *rbc*L and *tuf*A; Sciuto et al., 2015; Terlova and Lewis, 2019; Shetty et al., 2021). However, the phylogenetic relationships still remain unclear because of insufficient molecular data to construct a high-resolution phylogeny of the genus *Tetradesmus*. Furthermore, several molecular data (e.g., marker genes) in public databases were generated from morphologically unverified species (Mai et al., 2023). Such unverified sequences could cause confusions in interpreting the phylogenetic relationships of *Tetradesmus* species. Currently, genome sequencing information from diverse taxa is available because of the reduction of costs for genome sequencing approaches. In particular, the phylogenetic analysis using chloroplast genomes was useful to construct high-resolution evolutionary relationships (Lemieux et al., 2007; Lee et al., 2016; Song et al., 2016). Nonetheless, only three chloroplast genomes (NC_008101, CM007919, and MK514088) are available from the genus *Tetradesmus* (de Cambiaire et al., 2006; Starkenburg et al., 2017).

In this study, we generated complete chloroplast genomes from 9 *Tetradesmus* strains and analyzed the morphologies and phylogenetic relationships of *Tetradesmus* species. Based on our data, we provide a clear phylogenetic relationship and reestablish a complicated taxonomic history of the genus *Tetradesmus*, including diverse (e.g., unicellular, plane-arranged, and bundle-like) cellular types. We also newly report *T*. *obliquus* f. *rectilineare* H.S.Cho & J.M.Lee, f. nov., *T*. *obliquus* var. *spiraformis* H.S.Cho & J.M.Lee, var. nov., *T*. *dissociatus* f. *oviformis* H.S.Cho & J.M.Lee, f. nov., and *T*. *lancea* H.S.Cho & J.M.Lee, sp. nov. as novel formae, variety, and species in the genus *Tetradesmus*. Our results can provide a comprehensive understanding of taxonomic approaches for exploring the genus *Tetradesmus*.

## 2 Materials and methods

### 2.1 Algal strains and microscopic observation

The algal strains for genome sequencing were obtained from Freshwater Bioresources Culture Collection (FBCC) at the Nakdonggang National Institute of Biological Resources (Republic of Korea), Culture Collection of Algae and Protozoa (CCAP, UK), Culture Collection of Algae at the University of Texas (UTEX, USA), and Culture Collection of Algae at the University of Göttingen (SAG, Germany). All algal strains were cultured in 3 N Bold’s Basal Medium (Nichols and Bold, 1965) under 12:12 light/dark photocycle with a light intensity of 100 µmol·m^−2^·s^−1^ in a 20°C incubation chamber.

The morphological observation of *Tetradesmus* cells was performed under a light microscope (LM; ECLIPSE Ni-U, Nikon, Tokyo, Japan) and a scanning electron microscope (SEM; SU8220, Hitachi Ltd., Tokyo, Japan). To prepare samples for SEM observation, cells were treated with 2% glutaraldehyde solution for fixation and dehydrated through a series of ethanol concentrations (20%, 30%, 40%, 50%, 60%, 70%, 80%, 90%, and 100% ethanol) followed by critical-point drying (CPD; HCP-2, Hitachi Ltd., Tokyo, Japan). Then, the samples were coated with platinum. To measure the cell sizes (length and width) of *Tetradesmus* species, 100 cells were observed under the light microscope.

The illustrations of *Tetradesmus species* were drawn based on original descriptions and related literature (**Supplementary Table 1**; Bohlin, 1897; Kützing, 1834; Lagerheim, 1882; Lemmermann, 1898; Meyen, 1829; Turpin, 1828; Chodat, 1902; Reinhard, 1904; Collins, 1909; Chodat, 1913; Smith, 1913; Wołoszyńska, 1914; Printz, 1915; West, 1915; Smith, 1916; Chodat, 1926; Khristjuk, 1926; Huber-Pestalozzi, 1936; Hortobágyi, 1941; Prescott, 1944; Korshikov, 1953; Shen, 1956; Verses and Trainor, 1966; Fott and Komárek, 1974; Wang, 1990; Hu, 1992; Holtmann, 1994; Ettl and Gärtner, 1995; Hegewald and Hanagata, 2000; Wasser and Tsarenko, 2000; Tsarenko and Petlevanny, 2001; Lewis and Flechtner, 2004; Tsarenko et al., 2005; Hegewald et al., 2013; Wynne and Hallan, 2015; Wynne and Guiry, 2016; Lewis and Flechtner, 2019; Mikhailyuk et al., 2019; Taşkın, 2019; Terlova and Lewis, 2019).

### 2.2 DNA extraction, genome sequencing, assembly, gene prediction and annotation

Genomic DNA was extracted using the DNeasy Plant Mini Kit (Qiagen, Hilden, Germany) according to the manufacturer’s instructions. Genome sequencing data were generated using the NovaSeq6000 sequencing platform (Illumina, San Diego) with a 151–bp paired-end sequencing library. The sequencing raw reads were assembled using SPAdes Assembler (v3.14.2; Bankevich et al., 2012) and NOVOplasty (v4.3.1; Dierckxsens et al., 2017). Chloroplast genome contigs of *Tetradesmus* species were sorted by local-BLASTn search (*e*-value cutoff=1.*e*-05) with the chloroplast genome of *T*. *obliquus* (NC_008101). From the sorted contigs, complete chloroplast genome sequences were reassembled using Geneious Prime (v2022.2). The chloroplast genome of SAG 38.81 was reconstructed from the genome assembly (GCA_902809745.2; Astafyeva et al., 2020). The prediction of protein-coding genes in the chloroplast genomes was performed by BLASTx search (*e*-value cutoff=1.*e*-05; NCBI codon table 11) and annotated using Geneious Prime (v2022.2). Ribosomal RNA (rRNA) sequences (5S, 16S, and 23S) in the chloroplast genomes were predicted by local-BLASTn search (*e*-value cutoff=1.*e*-05) with rRNA sequences in the chloroplast genome of *T*. *obliquus* (NC_008101). Transfer RNA (tRNA) sequences were predicted using the ARAGORN program (Laslett and Canback, 2004).

### 2.3 Multigene phylogeny using chloroplast genomes

To construct a concatenated alignment of chloroplast genes, we used conserved 69 chloroplast protein sequences from 13 *Tetradesmus* and three outgroup species, and each homologous gene set was aligned using MAFFT (v7.450) under default options (Katoh and Standley, 2013). The maximum likelihood (ML) tree was constructed using 69 concatenated amino acid sequences (partition information with -q option) using the IQ-tree program (v1.6.12; Nguyen et al., 2015). The phylogenetic model was determined using the model test option (-m TEST), and ultrafast bootstrap search was conducted with 1,000 replications (-bb 1,000).

## 3 Results and discussion

### 3.1 General features of chloroplast genomes from *Tetradesmus* species

**Figure 1:**
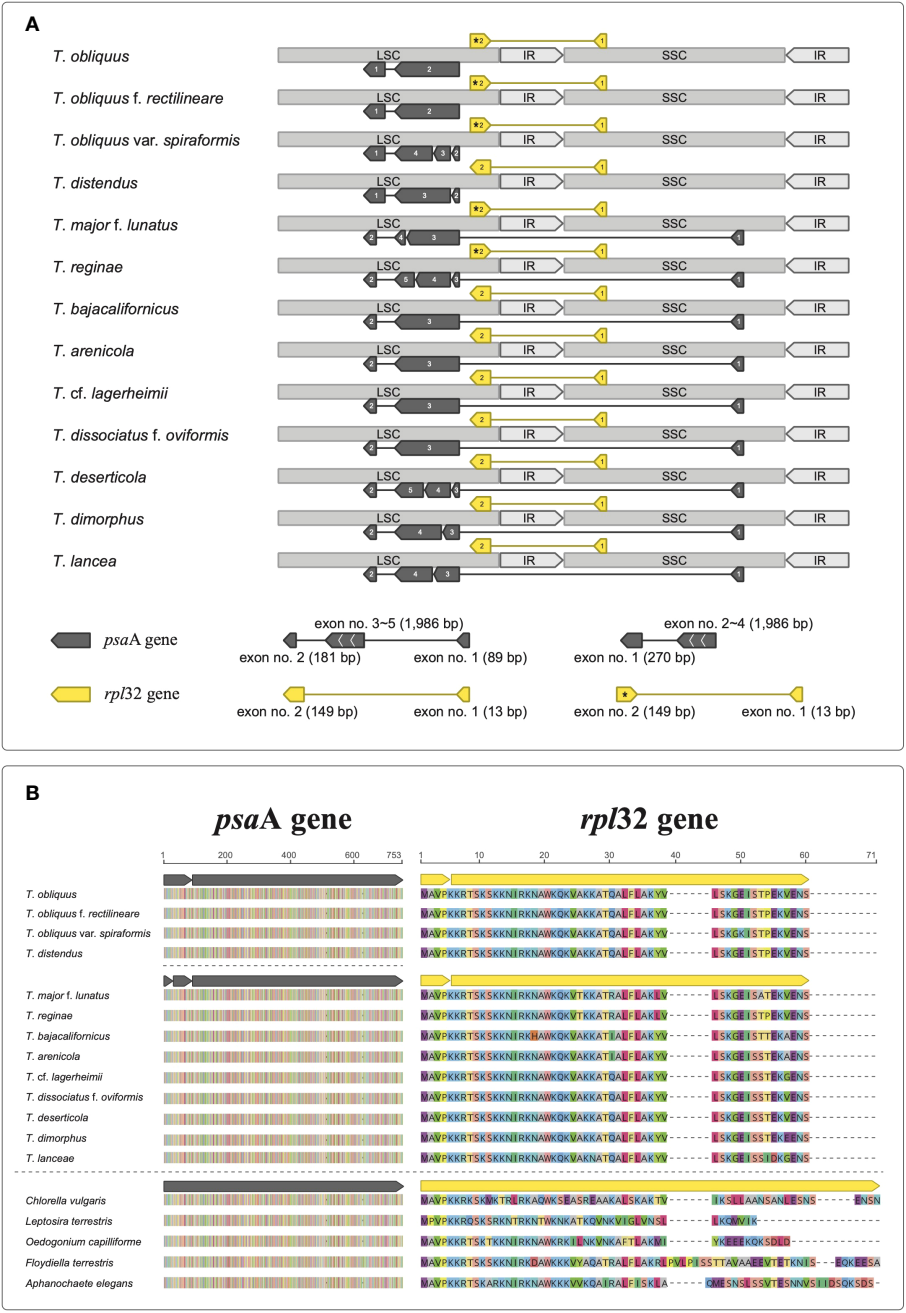
Exon-intron structures in the chloroplast genomes of *Tetradesmus* species. **(A)** Discontinuous exon structures of *psa*A and *rpl*32. The opposite direction of the second exon in *rpl*32 is indicated by asterisk. **(B)** Multiple sequence alignments of *psa*A and *rpl*32 from *Tetradesmus* species with the single-exon genes (*psa*A: NP_045850, QUO99137, YP_001382199, YP_003795480, and YP_009774535; *rpl*32: NP_045889, QUO99139, YP_001382159, YP_003795477, and YP_009774556).

We successfully constructed the circular chloroplast genomes of our target *Tetradesmus* strains (**Table 1**), which exhibited conserved genome structures and gene contents (CDS, rRNA, and tRNA) compared with the chloroplast genome (NC_008101) of *T*. *obliquus* f. *rectilineare* f. nov. UTEX 393 (detailed descriptions of novel forma are given in the next section). The chloroplast genomes of *Tetradesmus* species varied in size from 148,816 to 196,309 bp (**Table 1**). Interestingly, the *psa*A gene showed several different types of exon-intron boundaries and exon orders (**Figure 1A**). The exon orders of *psa*A in the chloroplast genomes of *Tetradesmus* species are roughly divided into two types as follows: the first exon is located in the large single copy (LSC) region or the small single copy (SSC) region (**Figure 1A**). In *T*. *obliquus* (Turpin) M.J.Wynne, *T*. *obliquus* f. *rectilineare* f. nov., *T*. *obliquus* var. *spiraformis* var. nov., and *T*. *distendus* (T.Holtmann) M.J.Wynne, the first exon of *psa*A is located in the downstream region (i.e., the last position) of its following exons on the LSC region (**Figure 1A**). The dipartite exon structure of *psa*A has been reported in the genus *Tetradesmus* (de Cambiaire et al., 2006). In another type from *Tetradesmus* species, the first exon is located in the SSC region, and the second exon is located in the downstream region of its following exons on the LSC region (**Figure 1A**). The discontinuous and (independently transcribed) tripartite exon structures of *psa*A have been reported in the CS clade (Chlamydomonadales and Sphaeropleales clade) of the class Chlorophyceae (Kück et al., 1987; Brouard et al., 2010; Fučíková et al., 2016; Watanabe et al., 2016). Although several types of exon order exist in *psa*A of *Tetradesmus* species, the amino acid sequences are highly conserved compared with those of single-exon *psa*A genes in green algal species from the non-CS clade (**Figure 1B**; NP_045850, YP_001382199, QUO99137, YP_003795480, and YP_009774535). All 13 *Tetradesmus* strains in this study contained the *rpl*32 gene, including two exons, with the first exon located in the SSC region and the second exon located in the LSC region (**Figure 1A**). However, the direction of the second exon in *rpl*32 is reversed in five *Tetradesmus* strains [*T*. *obliquus*, *T*. *obliquus* f. *rectilineare*, *T*. *obliquus* var. *spiraformis*, *T*. *major* f. *lunatus* (Korshikov) Fott & Komárek, and *T*. *reginae* (T.Holtmann) M.J.Wynne; asterisk in **Figure 1A**]. The amino acid sequences of *rpl*32 are highly conserved in the *Tetradesmus* species (**Figure 1B**). The discontinuous structure of *rpl32* has been previously reported in *Jenufa minuta* Nemcová, M.Eliás, Skaloud & Neustupa (Chlorophyceae, *incertae sedis*; Turmel et al., 2020). We postulate that diverse exon structures of *psa*A and *rpl*32 in the genus *Tetradesmus* could be derived from species-specific (or infraspecific) genetic variations. Moreover, *T*. *obliquus* UTEX 3031 and *T*. *obliquus* f. *rectilineare* UTEX 393 exhibit different exon-intron structures in *psb*A and *psa*B in the chloroplast genomes (**Supplementary Figure 1**). Therefore, the chloroplast genomes can provide information regarding genetic and structural variations for taxonomic identification at variety- or forma-level in the genus *Tetradesmus*.

**Table 1:** General features of chloroplast genomes in *Tetradesmus* species.

Species	Strain identifier	Genome size (bp)	CDS	rRNA	tRNA	GC %	Genbank accession
*Tetradesmus arenicola*	SAG 2564	151,685	69	6	30	26.6	OR502670
*Tetradesmus bajacalifornicus*	SAG 3.99	156,181	69	6	30	26.6	OR502669
*Tetradesmus deserticola*	BCP-SNI-2	171,548	69	6	30	25.7	MK514088
*Tetradesmus dimorphus*	FBCC-A330	196,309	69	6	30	28.4	OR502668
*T. dissociatus* f. *oviformis*	SAG 5.95	168,621	69	6	30	26.8	OR502667
*Tetradesmus distendus*	FBCC-A1020	167,760	69	6	30	30.0	OR502666
*Tetradesmus* cf. *lagerheimii*	SAG 38.81	184,761	69	6	30	30.1	See Method*
*Tetradesmus lanceae*	FBCC-A708	174,598	69	6	30	28.9	OR502671
*T*. *major* f. *lunatus*	FBCC-A1035	148,816	69	6	30	27.5	OR502665
*Tetradesmus obliquus*	UTEX 3031	167,272	69	6	30	27.0	KX756229
*T. obliquus* f. *rectilineare*	UTEX 393	161,307	69	6	30	26.9	NC_008101
*T. obliquus* var. *spiraformis*	SAG 22.81	179,208	69	6	30	27.1	OR502672
*Tetradesmus reginae*	CCAP 276/66	174,511	69	6	30	28.1	OR502664

*The chloroplast genome of SAG 38.81 was reconstructed from the genome assembly (GCA_902809745.2; Astafyeva et al., 2020).

### 3.2 Phylogenetic relationship and morphological description of *Tetradesmus* species

**Figure 2:**
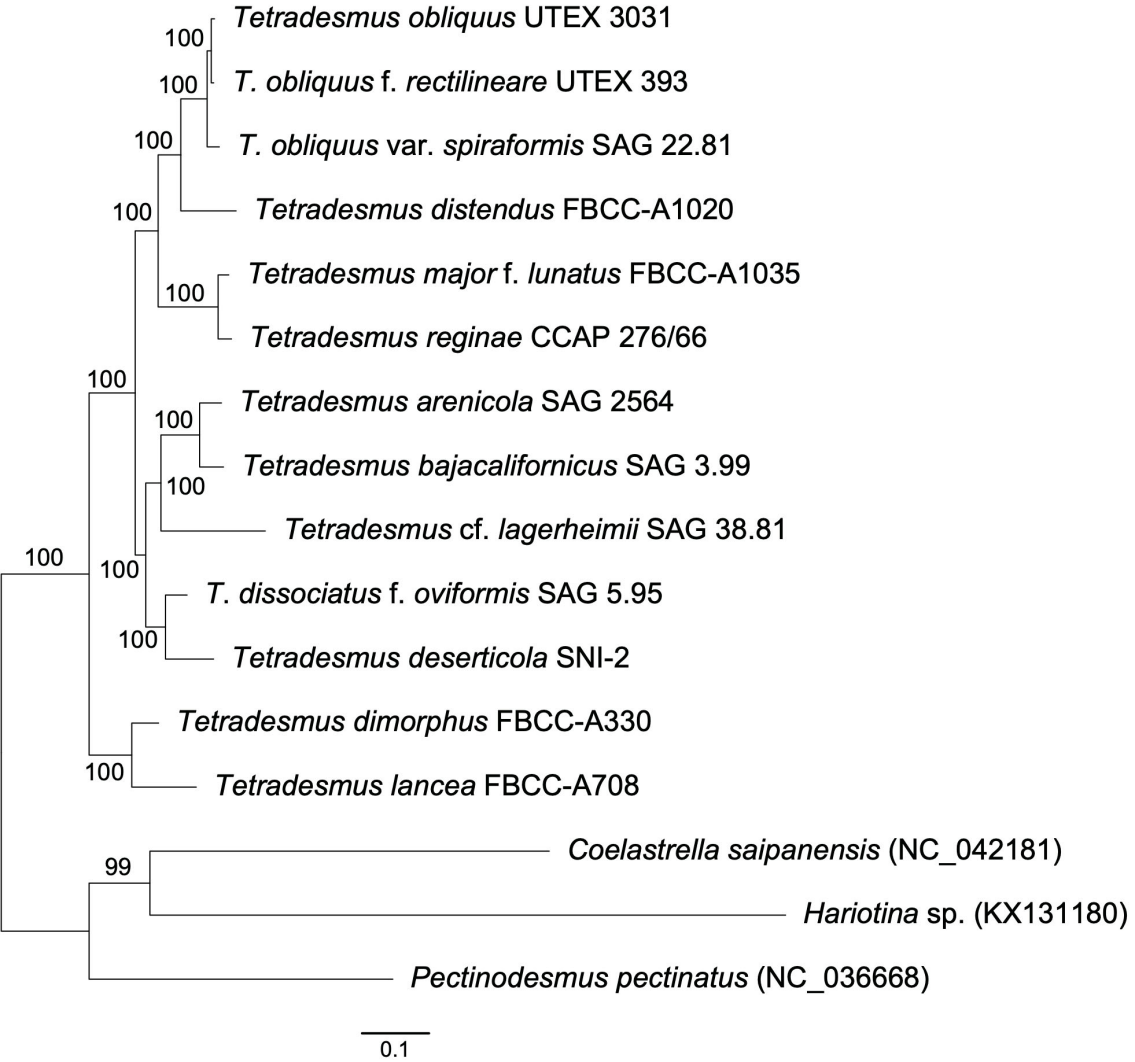
Maximum likelihood tree of *Tetradesmus* species constructed using a concatenated alignment of 69 chloroplast protein sequences (1,000 bootstrap replications).

**Figure 3:**
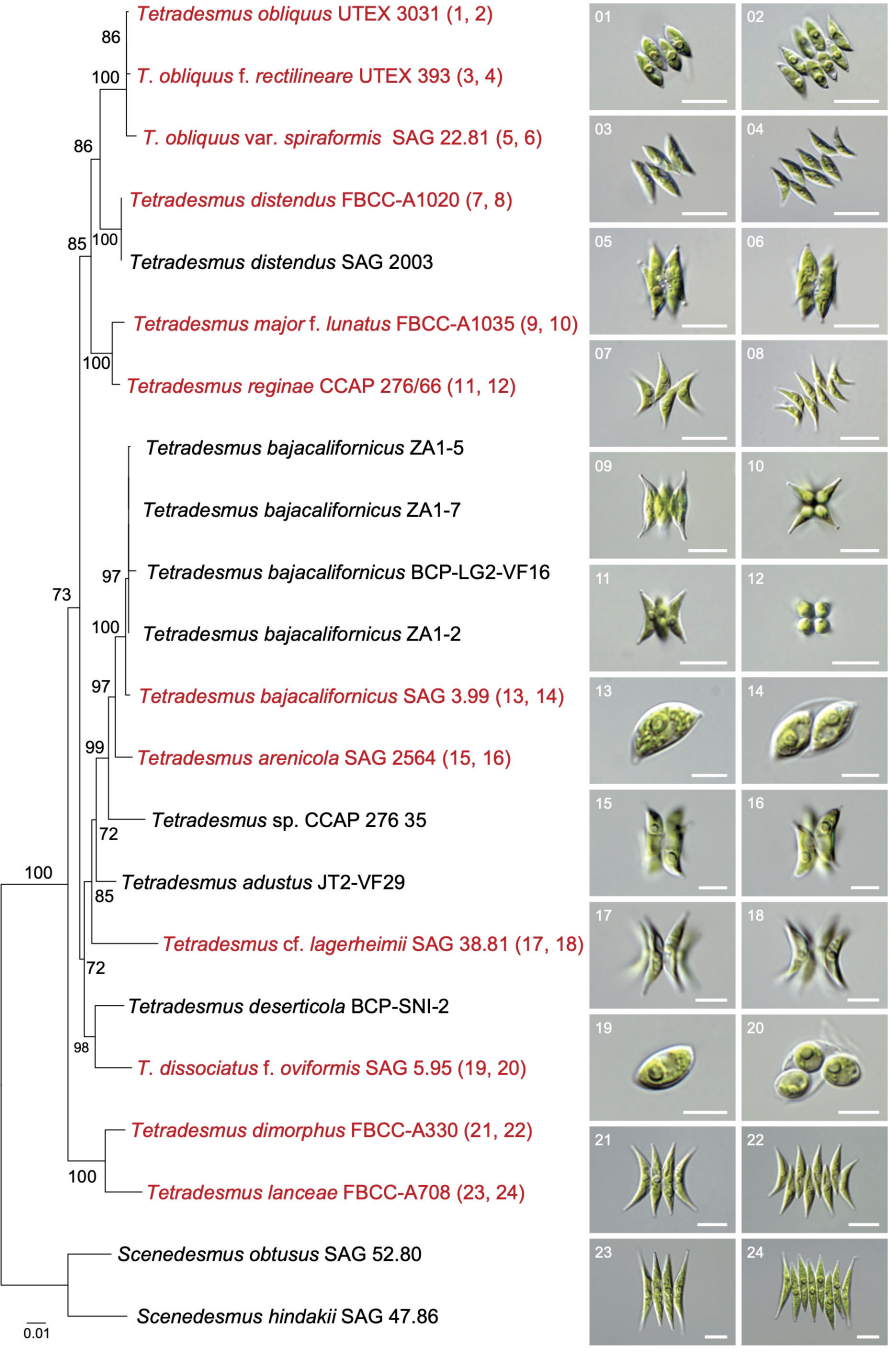
Phylogenetic analysis and microscopic observation of *Tetradesmus* species. The maximum likelihood tree was constructed using the ITS (ITS1-5.8S-ITS2) region, *rbc*L, and *tuf*A sequences (1,000 bootstrap replications; only >70% bootstrap supporting values are shown). Scale bars in the images of light microscopy indicate 10 µm (image no. 1–12, 15–16, and 21–24) and 5 µm (image no. 13–14 and 17–20).

To explore the phylogenetic relationship of *Tetradesmus* species, we conducted a multigene phylogeny using 69 conserved protein sequences found in the chloroplast genomes (**Figure 2**). The phylogenetic relationships of the 13 *Tetradesmus* strains are clearly addressed, and all nodes are fully supported (100% bootstrap supporting values; BS; **Figure 2**). In the multigene phylogeny, the early diverged clade is composed of *T*. *dimorphus* (Turpin) M.J.Wynne and *T*. *lanceae*, and the sister clade is divided into two monophyletic groups as follows: (group 1) *T*. *arenicola* Mikhailyuk & P.M.Tsarenko, *T*. *bajacalifornicus* L.A.Lewis & Flechtner, *T*. *lagerheimii* M.J.Wynne & Guiry, *T*. *dissociatus* f. *oviformis*, and *T*. *deserticola* L.A.Lewis & Flechtner; (group 2) *T*. *obliquus*, *T*. *obliquus* f. *rectilineare*, *T*. *obliquus* var. *spiraformis*, *T*. *distendus*, *T*. *major* f. *lunatus*, and *T*. *reginae* (**Figure 2**). In group 1, *T*. *arenicola* and *T*. *bajacalifornicus* show a monophyletic clade, and *T*. *lagerheimii* is their sister branch. *T*. *dissociatus* f. *oviformis* and *T*. *deserticola* are monophyly as a sister clade of the other taxa in group 1. In group 2, *T*. *obliquus* species exhibit a monophyletic group, and *T*. *obliquus* var. *spiraformis* SAG 22.81 is indicated as an early diverged taxon. The *T*. *obliquus* clade and *T*. *distendus* are monophyly, and their sister clade includes *T*. *major* f. *lunatus* and *T*. *reginae* (**Figure 2**). To investigate the phylogenetic relationship of *Tetradesmus* species with broad taxon samples, we constructed an ML tree (IQ-tree v1.6.12; Nguyen et al., 2015) using the nucleotide sequences of the ITS (ITS1-5.8S-ITS2) region and two chloroplast genes (*rbc*L and *tuf*A; **Table 2**, **Figure 3**). Although supporting values in several branches were relatively low, the phylogenetic tree constructed using the marker genes (**Figure 3**) could yield the same phylogenetic relationship of *Tetradesmus* species as that of the multigene phylogeny using the 69 chloroplast protein-coding genes (**Figure 2**).

**Table 2:** Taxon samples of *Tetradesmus* and *Scenedesmus* species for the phylogenetic analysis.

Species	Strain	Genbank accession		
		ITS1-5.8S-ITS2	*rbc*L	*tuf*A
*Scenedesmus hindakii*	SAG 47.86	AY170856	HG514369	HG514397
*Scenedesmus obtusus*	SAG 52.80	HG514421	HG514371	HG514400
*Tetradesmus adustus*	JT2-VF29	MK291427	MK291428	MK291429
*Tetradesmus arenicola*	SAG 2564	OR502670*	OR502670**	OR502670**
*Tetradesmus bajacalifornicus*	BCP-LG-VF16	AY510468	HQ246352	HQ246373
*Tetradesmus bajacalifornicus*	ZA1-2	HQ246448	HQ246354	HQ246374
*Tetradesmus bajacalifornicus*	ZA1-5	HQ246449	HQ246356	HQ246375
*Tetradesmus bajacalifornicus*	ZA1-7	HQ246450	HQ246357	HQ246376
*Tetradesmus bajacalifornicus*	SAG 3.99	OR502669*	OR502669**	OR502669**
*Tetradesmus deserticola*	BCP-SNI-2	AY510471	MK514088	MK514088
*Tetradesmus dimorphus*	FBCC-A330	OR502668*	OR502668**	OR502668**
*Tetradesmus dissociatus* f. *oviformis*	SAG 5.95	OR502667*	OR502667**	OR502667**
*Tetradesmus distendus*	FBCC-A1020	OR502666*	OR502666**	OR502666**
*Tetradesmus distendus*	SAG 2003	HG514429	HG514383	HG514411
*Tetradesmus* cf. *lagerheimii*	SAG 38.81	MK975480	See Method	See Method
*Tetradesmus lanceae*	FBCC-A708	OR502671*	OR502671**	OR502671**
*Tetradesmus major* f. *lunatus*	FBCC-A1035	OR502665*	OR502665**	OR502665**
*Tetradesmus obliquus*	UTEX 3031	GCA_002149895*	KX756229**	KX756229**
*Tetradesmus obliquus* f. *rectilineare*	UTEX 393	KP645233	NC_008101**	NC_008101**
*Tetradesmus obliquus* var. *spiraformis*	SAG 22.81	OR502672*	OR502672**	OR502672**
*Tetradesmus reginae*	CCAP 276/66	OR502664*	OR502664**	OR502664**
*Tetradesmus* sp.	CCAP 276/35	HG514426	HG514380	HG514408

*Only the ITS (ITS1-5.8S-ITS2) region from the rRNA sequences (18S-ITS1-5.8S-ITS2-28S partial) was used for the phylogenetic analysis.

**The nucleotide sequences of rbcL and tufA were used from the chloroplast genome sequences.

Although UTEX 393 and SAG 22.81 were previously labeled as *T*. *obliquus*, we suggest that these strains are a forma and a variety of *T*. *obliquus*, respectively, based on different exon-intron structures in the chloroplast genomes (i.e., *psa*A, *psa*B, and *psb*A; **Figure 1**, **Supplementary Figure 1**) and their distinct morphological characteristics (**Figure 3**). *T*. *obliquus* (basionym: *Achnanthes obliqua*) was characterized by spindle-shaped cells with acute apices, and their cells are typically arranged on a flat plane in two rows as four- or eight-celled coenobia (**Table 3**, **Figure 3**; Turpin, 1828; Smith, 1916). Especially, an eight-celled coenobium of *T*. *obliquus* (UTEX 3031) displays an oblique arrangement between each four-celled unit (**Figure 3**, **Supplementary Figure 2**), which was illustrated in their original description (Turpin, 1828). However, the morphological characteristics of UTEX 393 in the eight-celled coenobia exhibit rectilinear cellular arrangements in two rows (**Table 3**, **Figure 3**, **Supplementary Figure 2**). These strains also present distinct exon-intron structures (*psb*A and *psa*B; **Supplementary Figure 1**). Therefore, we propose that the distinct morphological characteristics and exon-intron structures between UTEX 3031 and UTEX 393 are regarded as irreversible infraspecific variations, specifically in exon-intron structures. Despite these distinctive features, several representative marker genes between UTEX 3031 and UTEX 393 remain conserved, with a few nucleotide differences (identical in 18S rRNA, 1 bp in ITS2, 5 bp in *rbc*L, and 3 bp in *tuf*A). Particularly, the most representative marker gene, 18S rRNA (commonly used for species identification), is identical in these strains, thus UTEX 393 should be considered as a novel forma of *T*. *obliquus*, specifically *T*. *obliquus* f. *rectilineare*. Furthermore, the cells of SAG 22.81 exhibit bundle-like and twisted coenobia (**Table 3**, **Figure 3**, **Supplementary Figure 2**). Previously, Hegewald and Hanagata (2000) and Sciuto et al. (2015) suggested SAG 22.81 as *T*. *reginae* and *T*. *obliquus*, respectively, but we propose that SAG 22.81 is *T*. *obliquus* var. *spiraformis* as a novel variety of *T*. *obliquus*. This proposal is based on genetic variations in marker genes (4 bp in 18S rRNA, 2 bp in ITS2, 10 bp in *rbc*L, and 8 bp in *tuf*A), exon-intron boundaries with exon orders (*psa*A; **Figure 1A**) in their chloroplast genomes, and distinct morphological differences (e.g., bundle-like coenobia) observed in SAG 22.81 compared to *T*. *obliquus* UTEX 3031.

**Table 3:** Morphological characteristics of *Tetradesmus* species.

Species	Strain	Cellular arrangement	Cells per coenobium	Length (µm)	Width (µm)	References
*Tetradesmus bajacalifornicus*	BCP-LG2-VF16	Unicelluar	Solitary	4.5 – 15.0	3.0 – 7.5	Lewis and Flechtner, 2019
	SAG 3.99			7.6 – 13.6 (avg. 10.6)	3.8 – 7.8 (avg. 5.8)	This study
*Tetradesmus dissociatus*	UTEX 1537	Unicelluar	Solitary	8.0 – 18.0	3.0 – 8.0	Verses and Trainor, 1966
*T*. *dissociatus* f. *oviformis*	SAG 5.95			6.8 – 12.4 (avg. 8.5)	3.6 – 8.3 (avg. 5.5)	This study
*Tetradesmus dimorphus*	FBCC-A330	Slightly curved plane in 1 or 2 rows	4 or 8 (rarely 2)	12.4 – 28.6 (avg. 20.4)	3.2 – 8.4 (avg. 5.3)	This study
	N/A	Flat plane (Smith, 1916) in 1 or 2 rows	2 or 4 or 8	6 – 25	2 – 9.4	Turpin, 1828; Smith, 1916; Tsarenko and John, 2011
*Tetradesmus lancea*	FBCC-A708	Slightly curved plane in 1 or 2 rows	4 or 8 (rarely 2)	21.7 – 38.2 (avg. 29.7)	3.0 – 8.0 (avg. 4.8)	This study
*Tetradesmus lagerheimii*	SAG 38.81	Strongly curved plane in 1 row	2 or 4 (mostly solitary)	11.4 – 19.2 (avg. 14.5)	2.4 – 5.1 (avg. 3.4)	This study
	N/A		4	30 – 40	6 – 7	Lagerheim, 1882
	N/A		4	30 – 50	6 – 7	Chodat, 1902
*Tetradesmus obliquus*	UTEX 3031	Flat plane in 1 or 2 rows	4 or 8 (rarely 2)	8.1 – 12.3 (avg. 10.2)	2.9 – 4.2 (avg. 3.6)	This study
	N/A	Flat plane in 1 or 2 rows	4 or 8	5.0 – 27.0	3.0 – 9.0	Turpin, 1828; Smith, 1916
*T. obliquus* f. *rectilineare*	UTEX 393	Flat plane in 1 or 2 rows	4 or 8 (rarely 2)	6.8 – 17.9 (avg. 13.8)	2.3 – 5.1 (avg. 3.8)	This study
*T. obliquus* var. *spiraformis*	SAG 22.81	Bundle-like	4 or 8 (rarely 2)	10.9 – 16.9 (avg. 13.2)	3.1 – 6.4 (avg. 4.1)	This study
*Tetradesmus distendus*	FBCC-A1020	Flat plane in 1 or 2 rows	4 or 8 (rarely 2)	12.4 – 23.5 (avg. 17.3)	3.2 – 8.4 (avg. 5.2)	This study
	SAG 2003	Flat plane in 1 or 2 rows	4 or 8 (rarely 2)	10.6 – 18.0 (avg. 14.3)	2.4 – 5.3 (avg. 3.6)	*This study
*Tetradesmus arenicola*	SAG 2564	Bundle-like	2 or 4 or 8 (mostly solitary)	19.4 – 29.4	7.8 – 8.3	Mikhailyuk et al., 2019
*Tetradesmus major* f. *lunatus*	FBCC-A1035	Bundle-like	4 or 8 (rarely 2)	12.4 – 22.7 (avg. 18.2)	3.2 – 6.7 (avg. 5.0)	This study
	N/A		4	17.0 – 18.0	3.0 – 5.0	Fott and Komárek, 1974
*Tetradesmus reginae*	CCAP 276/66	Bundle-like	4 or 8 (rarely 2)	8.7 – 13.9 (avg. 11.6)	2.1 – 5.0 (avg. 3.11)	This study
	N/A		4 or 8	10.0 – 11.5	2.2 – 3.5	Tsarenko et al., 2005

*The SAG 2003 strain was only used for measuring morphological data.

The detailed descriptions of *T*. *obliquus* f. *rectilineare* and *T*. *obliquus* var. *spiraformis* are given at the end of this section. The sister clade of *T*. *obliquus* strains is composed of *T*. *distendus* strains (FBCC-A1020 and SAG 2003), which possess similar morphological characteristics (e.g., cell shape and coenobial arrangement) as those of *T*. *obliquus* f. *rectilineare*; however, *T*. *distendus* displays more curved marginal cells and acute apices (**Table 3**, **Figure 3**, **Supplementary Figure 2**). The morphological characteristics of FBCC-A1035 correspond to the original description of *T*. *major* f. *lunatus* (Fott and Komárek, 1974), which typically exhibits arcuate and crescent-shaped cells (**Table 3**, **Figure 3**, **Supplementary Figure 2**). *T*. *major* f. *lunatus* (FBCC-A1035) has more curved cells with acute apices and a slightly larger cell size than that of *T*. *reginae* (CCAP 276/66; Hegewald et al., 2013), although these two species commonly display bundle-like coenobia (**Table 3**, **Figure 3**, **Supplementary Figure 2**).

The SAG 3.99 strain was previously labeled as *T*. *wisconsinensis* in the culture collection (Culture Collection of Algae at the University of Göttingen, SAG, Germany), which exhibits lemon-shaped unicells with an oval outline and pointed ends (**Figure 3**); however, *T*. *wisconsinensis* typically displays crescent-shaped cells and bundle-like coenobia (Smith, 1913). The morphological characteristics of SAG 3.99 correspond to the reference strain of *T*. *bajacalifornicus* LG2-VF16, which exhibits crescent- to lemon-shaped unicells with pointed ends and apical thickening (**Table 3**; Lewis and Flechtner, 2004; Lewis and Flechtner, 2019). Moreover, SAG 3.99 is closely clustered with *T*. *bajacalifornicus* strains (ZA1-2, ZA1-5, ZA1-7, and BCP-LG2-VF16) in the phylogenetic analysis (**Figure 3**). Hence, we suggest that SAG 3.99 can be considered *T*. *bajacalifornicus*. A sister branch of the *T*. *bajacalifornicus* clade is *T*. *arenicola* (SAG 2564 as the reference strain), which could exhibit lemon-shaped, crescent-shaped, or fusiform cells with pointed ends (**Figure 3**, **Supplementary Figure 2**). Furthermore, *T*. *arenicola* frequently forms both unicells and bundle-like coenobia with variation of cellular arrangements (Mikhailyuk et al., 2019). The sister branches of the clade, which includes *T*. *bajacalifornicus* and *T*. *arenicola*, are composed of *Tetradesmus* sp. CCAP 276/35 (BS 99%), *T*. *adustus* Terlova & L.A.Lewis (JT2-VF29; BS 72%), and *T*. *lagerheimii* SAG 38.81 (BS 85%; **Figure 3**). SAG 38.81 was labeled as *Scenedesmus acuminatus* (a synonym of *T*. *lagerheimii*) in the culture collection. SAG 38.81 displays crescent-shaped cells with acute apices and forms four-celled coenobia on a strongly curved plane (**Figure 3**, **Supplementary Figure 2**), which completely correspond to the typical morphological characteristics of *T*. *lagerheimii* (Lagerheim, 1882; Chodat, 1902). Nevertheless, the cells of SAG 38.81 are mostly solitary, and their cell size is considerably smaller than previous reports (**Table 3**). Therefore, we suggest that SAG 38.81 can be provisionally considered *T*. cf. *lagerheimii*, and hence further study is required to explore whether this strain is *T*. *lagerheimii* with cell size variation or a different *Tetradesmus* species. The sister clade of these taxa includes *T*. *deserticola* BCP-SNI-2 and *T*. *dissociatus* f. *oviformis* SAG 5.95 (**Figure 3**). SAG 5.95 was identified as *T*. *dissociatus* based on the comparison of ITS2 sequences with those of the reference strain, UTEX 1537 (Sciuto et al., 2015). However, the morphological characteristics of SAG 5.95 show ellipsoidal cells without apical thickening and pointed ends (**Table 3**, **Figure 3**). These characteristics are considerably different from the morphology of *T*. *dissociatus* UTEX 1537, particularly in the presence of long bridges at the pointed ends (**Supplementary Figures 3A, B**; Verses and Trainor, 1966). Moreover, the rRNA region of *T*. *dissociatus* UTEX 1537 contains intron sequences (784 bp; OR600236), which is distinct from SAG 5.95 (**Supplementary Figure 3C**), but 18S rRNA region is identical in these strains. Hence, we propose that SAG 5.95 is *T*. *dissociatus* f. *oviformis* as a novel forma of *T*. *dissociatus*. This proposal is based on genetic variations in 18S rRNA region (i.e., intron insertion) and morphological differences observed between SAG 5.95 and UTEXT1537.

The cells of FBCC-A330 are arranged on a slightly curved plane as four- and eight-celled coenobia, and the apices of marginal cells are curved (i.e., a crescent shape), in contrast to straight inner cells (**Figure 3**, **Supplementary Figure 2**). The cells of four-celled coenobia are arranged in one row, whereas the cells of eight-celled coenobia are arranged in two rows (**Figure 3**). The morphological characteristics of FBCC-A330 correspond to the original description of *T*. *dimorphus*, although there was no description of the plane arrangement (Turpin, 1828; Tsarenko and John, 2011). Smith (1916) described that the cells of *T*. *dimorphus* are arranged on a flat plane. However, we postulate that the illustrations indicate a slightly curved plane because their two marginal cells in the four-celled coenobia are arranged slightly ahead (or behind) of the inner cells (Smith, 1916), similar to that in FBCC-A330 (**Figure 3**, **Supplementary Figure 2**). Therefore, we identified FBCC-A330 as *T*. *dimorphus*. Nevertheless, several different types of sequences, which are labeled as *T*. *dimorphus*, were reported on the NCBI database, and most of them are unpublished data without morphological descriptions (i.e., morphologically unverified species). To compare our result and *T*. *dimorphus*-related sequences, we constructed an ML tree using the rRNA sequences (18S-ITS1-5.8S-ITS2 region) of *T*. *dimorphus* FBCC-A330 and their homologous sequences (BLASTn search *e*-value cutoff=1.*e*-10; **Supplementary Figure 4**; IQ-tree v1.6.12; Nguyen et al., 2015). Although no monophyletic taxon with *T*. *dimorphus* FBCC-A330 was observed in the ML tree, the cells of the four-celled coenobia of all previously reported *T*. *dimorphus*, which were morphologically described (green color in **Supplementary Figure 4**), are arranged in two rows (Ahamefule et al., 2018; Alam et al., 2019; Sehgal et al., 2019; Khaw et al., 2020; Chanu et al., 2021; Huang et al., 2022). The one-row arrangement in the four-celled coenobia is the distinguishing morphological characteristics of *T*. *dimorphus* (Turpin, 1828; Smith, 1916; Tsarenko and John, 2011). Thus, we suggest that, to date, only FBCC-A330 is morphologically verified *T*. *dimorphus*, corresponding to their morphological descriptions (Turpin, 1828; Smith, 1916; Tsarenko and John, 2011). The morphological characteristics of FBCC-A708 are similar to those of *T*. *dimorphus* FBCC-A330, but FBCC-A708 has longer cells and slightly less curved marginal cells (**Table 3**, **Figure 3**). Furthermore, both the four- and eight-celled coenobia in FBCC-A708 are arranged on a slightly curved plane in slightly two rows (**Figure 3**). Hence, we propose that FBCC-A708 is *T*. *lancea* as a novel species in the genus *Tetradesmus*.

*Tetradesmus obliquus* f. *rectilineare* H.S.Cho & J.M.Lee f. nov.

Description: Cells are spindle-shaped with acute apices. The cells of four- and eight-celled coenobia are arranged on a flat plane in two rows. The rectilinear cellular arrangement in two rows in the eight-celled coenobium is a significant morphological difference compared with that in *T*. *obliquus*. The length of cells is 6.8–17.9 μm (average 13.8 μm), and their width is 2.3–5.1 μm (average 3.8 μm). The cells contain one pyrenoid each (**Figure 3**, **Supplementary Figure 2**).

Holotype: The permanent slide (NNIBRCL23411) of strain UTEX 393 is deposited in the Freshwater Bioresources Culture Collection (FBCC; https://fbp.nnibr.re.kr/fbcc/) at Nakdonggang National Institute of Biological Resources, Sangju, Gyeongsangbuk-do, Republic of Korea.

Reference strain: UTEX 393 from the Culture Collection of Algae at the University of Texas (UTEX, USA).

Etymology: The specific epithet “*rectilineare*” is derived from the Latin word “*rectilineare*” (rectilinear), which indicates the morphological characteristics of the cellular arrangement in the eight-celled coenobium.

Type locality and habitat: Freshwater (the locality is not available).

Representative DNA sequence: rRNA sequences (18S rRNA partial, ITS1, 5.8S rRNA, ITS2, and 28S rRNA partial; KP645233) and chloroplast genome (NC_008101).

*Tetradesmus obliquus* var. *spiraformis* H.S.Cho & J.M.Lee var. nov.

Description: Cells are spindle-shaped with acute apices. Four- or eight-celled and twisted bundle-like coenobia are typical, which indicates a significant morphological difference compared with that in *T*. *obliquus*. The length of the cells is 10.9–16.9 μm (average 13.2 μm), and their width is 3.1–6.4 μm (average 4.1 μm). The cells contain one pyrenoid each (**Figure 3**, **Supplementary Figure 2**).

Holotype: The permanent slide (NNIBRCL23412) of strain SAG 22.81 is deposited in the Freshwater Bioresources Culture Collection (FBCC; https://fbp.nnibr.re.kr/fbcc/) at Nakdonggang National Institute of Biological Resources, Sangju, Gyeongsangbuk-do, Republic of Korea.

Reference strain: SAG 22.81 from the Culture Collection of Algae at the Georg-August-University Göttingen (Göttingen, Germany).

Etymology: The specific epithet “*spiraformis*” is derived from a compound word of the Latin words “*spiralis*” (spiral) and “*-formis*” (-form), which indicate the morphological characteristics of the coenobia.

Type locality and habitat: Freshwater, Laguna Pataccocha, Apurimac, Peru (13°44’06.74”S, 73°06’43.97”W).

Representative DNA sequence: rRNA sequences (18S rRNA, ITS1, 5.8S rRNA, ITS2, and 28S rRNA; OR530173) and chloroplast genome (OR502672).

*Tetradesmus dissociatus* f. *oviformis* H.S.Cho & J.M.Lee f. nov.

Description: Cells are oval or ellipsoidal in shape with pointed ends. The length of cells is 6.8–12.4 μm (average 8.5 μm), and their width is 3.6–8.3 μm (average 5.5 μm). The cells contain one pyrenoid each (**Figure 3**, **Supplementary Figure 2**).

Holotype: The permanent slide (NNIBRCL23413) of strain SAG 5.95 is deposited in the Freshwater Bioresources Culture Collection (FBCC; https://fbp.nnibr.re.kr/fbcc/) at Nakdonggang National Institute of Biological Resources, Sangju, Gyeongsangbuk-do, Republic of Korea.

Reference strain: SAG 5.95 from the Culture Collection of Algae at the Georg-August-University Göttingen (Göttingen, Germany).

Etymology: The specific epithet “*oviformis*” is derived from a compound word of the Latin words “*ovi*” (egg) and “*-formis*” (-form), which indicate the morphological characteristics of the cell outline.

Type locality and habitat: Freshwater, Bordeaux, France (the detailed locality is not available).

Representative DNA sequence: rRNA sequences (18S rRNA, ITS1, 5.8S rRNA, ITS2, and 28S rRNA; OR530172) and chloroplast genome (OR502667).

*Tetradesmus lancea* H.S.Cho & J.M.Lee sp. nov.

Description: Spindle-shaped inner and slightly curved marginal cells with typical acuminate apices. The cells of four-celled coenobia are arranged on a slightly curved plane in one row, and those of eight-celled coenobia are arranged in slightly two rows. The marginal cells of coenobia are generally longer than the inner cells. The length of cells is 21.7–38.2 μm (average 29.7 μm), and their width is 3.0–8.0 μm (average 4.8 μm). The cells contain one pyrenoid each (**Figure 3**, **Supplementary Figure 2**).

Holotype: The permanent slide (NNIBRCL19693) of strain FBCC-A708 is deposited in the Freshwater Bioresources Culture Collection (FBCC; https://fbp.nnibr.re.kr/fbcc/) at Nakdonggang National Institute of Biological Resources, Sangju, Gyeongsangbuk-do, Republic of Korea.

Reference strain: FBCC-A708 from FBCC at the Nakdonggang National Institute of Biological Resources (Republic of Korea).

Etymology: The specific epithet “*lancea*” is derived from the Latin word “*lancea*” (a light spear or lance), which indicates the morphological characteristics of the cell outline.

Type locality and habitat: Riverside, Gapyeong-gun, Gyeonggi-do, Republic of Korea (37°48′54.7′′N, 127°31′17.3′′E); freshwater.

Representative DNA sequence: rRNA sequences (18S rRNA, ITS1, 5.8S rRNA, ITS2, and 28S rRNA; OR530171) and chloroplast genome (OR502671).

### 3.3 Coenobial types of the genus *Tetradesmus*

**Figure 4:**
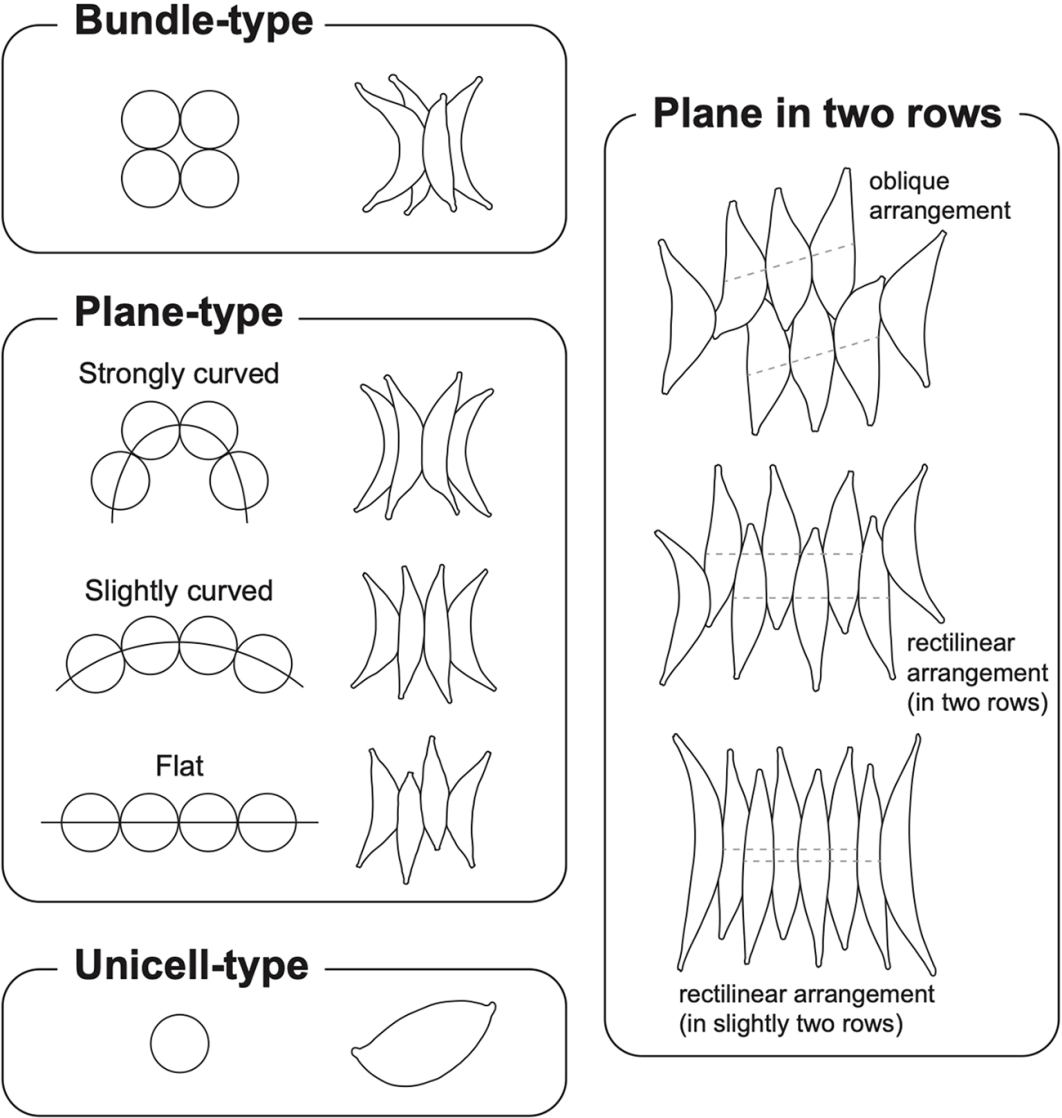
Coenobial types and cellular arrangements in *Tetradesmus* species.

The genus *Tetradesmus* was initially established based on the bundle-like coenobia of spindle-shaped cells (Smith, 1913). However, *T. wisconsinensis* (the type species of *Tetradesmus*) and several *Scenedesmus*-like species were integrated into the genus *Acutodesmus* (Tsarenko and Petlevanny, 2001; Hegewald et al., 2013); hence, this genus was composed of species with different types of cellular arrangements such as unicells, bundle-like, and plane-type (**Supplementary Figure 5**). Nonetheless, most *Acutodesmus* taxa, including *A*. *wisconsinensis*, were transferred to the genus *Tetradesmus* again because the genus *Acutodesmus* established in 2001, which occurred after the initial report of the type species *T*. *wisconsinensis* in 1913 (**Supplementary Figure 5**; Wynne and Hallan, 2015; Wynne and Guiry, 2016; Lewis and Flechtner, 2019). Here, we revisited the morphological characteristics of cellular arrangements in the genus *Tetradesmus* and clarified three types (i.e., bundle-type, plane-type, and unicell-type) of coenobial/cellular arrangements (**Figure 4**).

The representative coenobial type of the genus *Tetradesmus* is bundle-type (**Figure 4**), which corresponds to the coenobial formation of the type species *T*. *wisconsinensis* (Smith, 1913). The bundle-type coenobia are also present in *T*. *acuminatus*, *T*. *arenicola*, *T*. *cumbricus* G.S.West, *T*. *formosanus* Shen, *T*, *major* (Fischer) Fott & Komárek, *T*. *obliquus* var. *spiraformis*, *T*. *reginae*, and *T*. *smithii* Prescott (West, 1915; Prescott, 1944; Shen, 1956; Fott and Komárek, 1974; Hu, 1992; An et al., 1999; Mikhailyuk et al., 2019). The plane-type coenobia are divided into three categories (i.e., strongly curved, slightly curved, and flat plane) based on the degree of curve in the four-celled coenobial arrangements (**Figure 4**). For instance, the coenobial cells of *T*. *lagerheimii* are arranged on a strongly curved plane, and those of *T*. *dimorphus* and *T*. *lancea* are arranged on a slightly curved plane. Coenobial cells arranged on a flat plane are present in *T*. *bernardii* (G.M.Smith) M.J.Wynne, *T*. *distendus*, *T*. *nygaardii* (Huber-Pestalozzi) M.J.Wynne, *T*. *obliquus*, and *T*. *obliquus* f. *rectilineare* (Turpin, 1828; Smith, 1916; Huber-Pestalozzi, 1936; Holtmann, 1994). The two-row or slightly two-row arrangement (i.e., almost one line as in *T*. *lancea*) of the plane-type cells is recognized by arrangements of the inner cells (**Figure 4**). Moreover, the coenobial cells in two rows could exhibit oblique (e.g., *T*. *obliquus*) and rectilinear (e.g., *T*. *dimorphus* and *T*. *obliquus* f. *rectilineare*) arrangements (**Figure 4**). The unicell-type cells are typically present in *T*. *adustus*, *T*. *bajacalifornicus*, *T*. *deserticola*, *T*. *dissociatus*, and *T*. *dissociatus* f. *oviformis* (Verses and Trainor, 1966; Lewis and Flechtner, 2004; Lewis and Flechtner, 2019; Terlova and Lewis, 2019).

Although diverse coenobial types are present in the genus *Tetradesmus*, this trait is not phylogenetically conserved; hence, coenobial formations independently diverged irrespective of their evolutionary relationships (**Figures 2**, **3**). For instance, bundle-like coenobia are present in *T*. *obliquus* var. *spiraformis*, *T*. *major* f. *lunatus*, *T*. *reginae*, and *T*. *arenicola*, but most of their closely related taxa show unicell-type (e.g., *T*. *bajacalifornicus*) or plane-type (e.g., *T*. *obliquus* and *T*. *distendus*) cellular arrangements except *T*. *major* f. *lunatus* and *T*. *reginae* (**Figures 2**, **3**). Therefore, we postulate that the morphological characteristics of the coenobial types are generally species/variety/forma-specific features, which is suitable for taxonomic identifiers in the genus *Tetradesmus*, rather than for evolutionary interpretations such as morphological differentiations based on phylogenetic relationships.

### 3.4 Taxonomic issues in the genus *Tetradesmus*

Although we reinvestigated the taxonomy of *Tetradesmus* species based on morphological characteristics and chloroplast genomes, we recognized that potential taxonomic issues in several reports remain, as described in the following cases. For instance, *T*. *almeriensis* Turiel, Garrido-Cardenas, Gómez-Serrano, Acién, Carretero-Paulet & S.Blanco (the reference strain CCAP 276/24), which was reported as a new species in the genus *Tetradesmus*, displays oval or ellipsoidal cells without acuminate cell poles rather than spindle cells (Turiel et al., 2021). The oval or ellipsoidal cells are typical traits of the genus *Scenedesmus* (Tsarenko and John, 2011). Furthermore, the phylogenetic analysis using *rbcL* and ITS sequences revealed that *T*. *almeriensis* shows a monophyletic relationship with *Scenedesmus* species (Turiel et al., 2021). We confirmed the same phylogenetic relationship using ITS1-5.8S-ITS2, *rbc*L, and *tuf*A genes (**Supplementary Figure 6**); hence, we suggest transferring *T*. *almeriensis* CCAP 276/24 to the genus *Scenedesmus* as follows.

*Scenedesmus almeriensis* (Turiel, Garrido-Cardenas, Gómez-Serrano, Acién, Carretero-Paulet & S.Blanco) H.S.Cho & J.M.Lee comb. nov.

Basionym: *Tetradesmus almeriensis* Turiel, Garrido-Cardenas, Gómez-Serrano, Acién, Carretero-Paulet & S.Blanco, Processes 9(11): 2006. 2021.

Several *Tetradesmus* strains were previously labeled (e.g., UTEX 393 and SAG 22.81) as *T*. *obliquus* (or its synonym *Scenedesmus obliquus*) without accurate taxonomic and molecular investigations. For example, Lürling (2003) suggested that *T*. *obliquus* show a high degree of phenotypic plasticity among *T*. *obliquus* strains, including UTEX 393, but this *Tetradesmus* strain is newly identified as a different forma *T*. *obliquus* f. *rectilineare* in our study based on their distinct morphological characteristics. Specifically, we found significant genetic variations in exon-intron structures and exon order in the chloroplast genome of *T*. *obliquus* f. *rectilineare* UTEX393 compared to *T*. *obliquus* UTEX 3031, but we defined these differences as infraspecific variation, given the identical sequences of their 18S rRNA regions. The phenotypic plasticity in various *T*. *obliquus* strains requires further study to determine whether it is reversible or irreversible, both within a strain and between strains, in terms of morphological variations. In addition, it is essential to address how many genetic variations accumulate and their significance (e.g., nucleotide level, intron insertion, exon-intron structure, and exon order). Moreover, the investigation should explore how many similar or identical genetic variations can be identified among infraspecific taxa. We suggest that a more diverse analysis of genetic variations in chloroplast genomes among infraspecific taxa could address these points (or establish a fundamental background for further steps). Furthermore, this approach is more efficient than the analysis of nuclear genomes. As another aspect of morphological variations, several types of morphological changes (e.g., the number of colonial cells) are generally possible in diverse algal culture strains, including *Tetradesmus* species, depending on their cell division progression and culture conditions. We recommend that the identification of *Tetradesmus* species, when the target shows coenobial formation, is carried out based on four- or eight-celled coenobia derived from culture conditions, which should not typically exceed 2 weeks after subculture, with sufficient nutrients. To comprehensively verify the phenotypic plasticity within the genus *Tetradesmus*, further studies are required, including detailed morphological observations and culture experiments across diverse *Tetradesmus* strains that are correctly identified by both morphological and molecular evidence.

Several taxonomically accepted *Tetradesmus* species were described based on only morphological characteristics with no molecular data as described below. To review the morphological characteristics of *Tetradesmus* species, we redrew the illustrations of *Tetradesmus* species based on original descriptions and related literature (**Supplementary Figure 7**; references used for the illustrations are described in **Supplementary Table 1** and the Methods section). Most *Tetradesmus* cells are spindle-shaped with diverse cellular types (unicell, plane-type, and bundle-type), but the cells of *T*. *smithii* and *T*. *obliquus* var. *flexuosus* (Lemmermann) Taşkin & Alp show exceptional morphological features in the genus *Tetradesmus*. For instance, *T*. *smithii* exhibits a bundle-like coenobium, but its cells have rounded apices (Prescott, 1944). *T*. *obliquus* var. *flexuosus* exhibits oval cells as a chain-like formation (Lemmermann, 1898; Collins, 1909; Taşkın, 2019), which is completely different from the typical traits of the genus *Tetradesmus* (**Supplementary Table 1**, **Supplementary Figure 7**). Nevertheless, we cannot clearly determine whether these taxa have exceptional morphologies within the genus *Tetradesmus* or belong to other taxonomic groups because there is no available strain or sequence data from these taxa. Therefore, in further studies, molecular evidence (e.g., marker genes and chloroplast genomes) will be useful to identify and compare *Tetradesmus* species/varieties/formae, including the diverse morphological characteristics that could cause confusions with other taxonomic groups.

## 4 Conclusion

We provide a clear phylogenetic relationship of *Tetradesmus* species using chloroplast genomes and report a novel species, a novel variety, and two novel formae from this genus based on morphological characteristics such as cellular arrangements and exon-intron structures of the chloroplast genes. Our results and approaches will provide a comprehensive taxonomic understanding of the genus *Tetradesmus* and help identify cryptic species or variety from this genus. As interest in a wide range of applications using *Tetradesmus* species (e.g., *T*. *obliquus*) increases, our study will also help clarify species identification for biotechnological studies (Escorsim et al., 2018; Talarek-Karwel et al., 2020; Gouveia et al., 2021; Oliveira et al., 2021).

## Data availability statement

The data presented in the study are deposited in the NCBI database, accession numbers OR502664 - OR502672.

## Author contributions

HC: Data curation, Formal analysis, Investigation, Methodology, Software, Validation, Visualization, Writing – original draft, Writing – review & editing. JL: Conceptualization, Funding acquisition, Project administration, Resources, Supervision, Validation, Writing – original draft, Writing – review & editing.
