# Supplementary material for: Taxonomic reinvestigation of the genus Tetradesmus (Scenedesmaceae; Sphaeropleales) based on morphological characteristics and chloroplast genomes
Source: Front Plant Sci. 2024 Feb 14;15:1303175. doi: 10.3389/fpls.2024.1303175 (PMC10899504; doi:10.3389/fpls.2024.1303175)
Supplement: Supplementary file 1 [file Table_1.docx]

Supplementary Material

# Supplementary Figures and Table

## Supplementary Figures

**Supplementary Figure 1.** Exon structures of *psbA* and *psaB* in *T*. *obliquus* strains (UTEX 393 and UTEX 3031).

**Supplementary Figure 2.** Scanning electron microscopic images of *Tetradesmus* strains (scale bars: 3 µm). (A) *T*. *obliquus* UTEX 3031; (B) *T*. *obliquus* f. *rectilineare* UTEX 393; (C) *T*. *obliquus* var. *spiraformis* SAG 22.81; (D) *T*. *distendus* FBCC-A1020; (E) *T*. *major* f. *lunatus* FBCC-A1035; (F) *T*. *reginae* CCAP 276/66; (G) *T*. *bajacalifornicus* SAG 3.99; (H) *T*. *arenicola* SAG 2564; (I) *T*. cf. *lagerheimii* SAG 38.81; (J) *T*. *dissociatus* f. *oviformis* SAG 5.95; (K) *T*. *dimorphus* FBCC-A330; (L) *T*. *lancea* FBCC-A708.

**Supplementary Figure 3.** *Tetradesmus* *dissociatus* UTEX 1537. (A) Light microscopic images of *T*. *dissociatus* UTEX 1537. The bridge-like structure (arrow) of the cell is indicated by an arrowhead (scale bars = 5 µm). (B) Multiple sequence alignment of the rRNA regions in UTEX 1537 (AY510466, Lewis and Flechtner, 2004; OR600236 from this study) and SAG 5.95 (OR530172).

**Supplementary Figure 4.** The maximum likelihood tree of *Tetradesmus dimorphus* and related taxa constructed using ribosomal RNA (18S-ITS1-5.8S-ITS2) regions (only >50% bootstrap supporting values are shown). The sequences of *T*. *dimorphus* are highlighted by three colors as follows: red = sequence data only; green = available both sequence and morphological data; blue = *T*. *dimorphus* from this study.

**Supplementary Figure 5.** Taxonomic history of the genus *Tetradesmus*.

**Supplementary Figure 6.** The maximum likelihood tree of *Tetradesmus* (*Scenedesmus*) *almeriensis* (ITS, MF977406; *rbc*L, MG257492) and related taxa constructed using the ITS (ITS1-5.8S-ITS2) region, *rbc*L, and *tuf*A sequences (only >60% bootstrap supporting values are shown). Outgroup taxa are *Desmodesmus* *abundans* (ITS, OP103755; *rbc*L and *tuf*A from NC_066651) and *Desmodesmus communis* (*rbc*L, HG514364; *tuf*A, HG514391).

**Supplementary Figure 7.** Illustrations of *Tetradesmus* taxa based on original descriptions (Supplementary Table 1) and morphological observations of culture strains (this study).

**1.2 Supplementary Table 1**

**Supplementary Table 1.** Current taxonomy of the genus *Tetradesmus*.

| **Current Nomenclature** | | **Synonym** | **Reference** |  |
| --- | --- | --- | --- | --- |
|  |  |  |  |  |
| 1 | *T*. *acuminatus* C. –C. Jao & Z. –Y. Hu |  | Hu, 1992 |  |
| 2 | *T*. *adustus* Terlova & L. A. Lewis |  | Terlova and Lewis, 2019 |  |
| 3 | *T*. *arenicola* Mikhailyuk & P. Tsarenko |  | Mikhailyuk et al., 2019 |  |
| 4 | *T*. *bajacalifornicus* L.A.Lewis & Flechtner | *Scenedesmus bajacalifornicus* L.A.Lewis & Flechtner | Lewis and Flechtner, 2004, 2019; Hegewald et al., 2013 |  |
|  |  | *Acutodesmus bajacalifornicus* E.Hegewald, C.Bock & Krienitz |  |  |
| 5 | *T*. *bernardii* (G. M. Smith) M. J. Wynne | *Scenedesmus bernardii* G. M. Smith | Smith, 1916; Korshikov, 1953; Tsarenko and Petlevanny, 2001; Hegewald et al., 2013; Wynne and Hallan, 2015 |  |
|  |  | *Scenedesmus acuminatus* var. *bernardii*(G. M. Smith) Dedusenko |  |  |
|  |  | *Acutodesmus pectinatus* var. *bernardii* (G. M. Smith) P. M. Tsarenko |  |  |
|  |  | *Acutodesmus bernardii*(G. M. Smith) E. Hegewald, C. Bock & Krienitz |  |  |
| 6.1 | *T*. *cumbricus* G. S. West |  | West, 1915 |  |
| 6.2 | *T*. *cumbricus* var. *apiculatus* Korshikov |  | Korshikov, 1953 |  |
| 6.3 | *T*. *cumbricus* var. *gracilis* S. S. Wang |  | Wang, 1990 |  |
| 7 | *T*. *deserticola* L. A. Lewis & Flechtner | *Scenedesmus deserticola* L.A.Lewis & Flechtner | Lewis and Flechtner, 2004, 2019; Hegewald et al., 2013 |  |
|  |  | *Acutodesmus deserticola* E.Hegewald, C.Bock & Krienitz |  |  |
| 8 | *T*. *dimorphus* (Turpin) M. J. Wynne | *Achnanthes dimorpha* Turpin | Turpin, 1828; Kützing, 1834; Tsarenko and Petlevanny, 2001; Wynne and Hallan, 2015 |  |
|  |  | *Scenedesmus dimorphus* (Turpin) Kützing |  |  |
|  |  | *Acutodesmus dimorphus* (Turpin) P.M.Tsarenko |  |  |
| 9.1 | *T*. *dissociatus* (P. A. Verses & F. R. Trainor) M. J. Wynne | *Dactylococcus dissociatus* P. A. Verses & F. R. Trainor | Verses and Trainor, 1966; Ettl and Gärtner, 1995; Hegewald and Hanagata, 2000; Hegewald et al., 2013; Wynne and Hallan, 2015 |  |
|  |  | *Keratococcus dissociatus* (P. A. Verses & F. R. Trainor) H. Ettl & G. Gärtner |  |  |
|  |  | *Scenedesmus dissociatus* (P. A. Verses & F. R. Trainor) E. Hegewald & N. Hanagata |  |  |
|  |  | *Acutodesmus dissociatus* (P.A.Verses & F.R.Trainor) E. Hegewald, C. Bock et Krienitz |  |  |
| 9.2 | *T*. *dissociats* f. *oviformis* **f. nov.** H. S. Cho & J. M. Lee |  | This study |  |
| 10 | *T*. *distendus* (T. Holtmann) M. J. Wynne | *Scenedesmus pectinatus* var. *distentus*T. Holtmann | Holtmann, 1994; Hegewald and Hanagata, 2000; Hegewald et al., 2013; Wynne and Hallan, 2015 |  |
|  |  | *Scenedesmus distentus* (T. Holtmann) E. Hegewald & N. Hanagata |  |  |
|  |  | *Acutodesmus distendus* (T. Holtmann) E. Hegewald, C. Bock & Krienitz |  |  |
| 11 | *T*. *formosanus* Shen |  | Shen, 1956 |  |
| 12 | *T*. *hupehensis* Z. –Y. Hu |  | Hu, 1992 |  |
| 13 | *T*. *incrassatulus* (Bohlin) M.J.Wynne | *Scenedesmus incrassatulus* Bohlin | Bohlin, 1897; Tsarenko and Petlevanny, 2001; Wynne and Hallan, 2015 |  |
|  |  | *Acutodesmus incrassatulus* (Bohlin) P. M. Tsarenko |  |  |
| 14.1 | *T*. *lagerheimii* (Lagerheim) M.J.Wynne & M. D. Guiry | *Selenastrum acuminatum* Lagerheim | Lagerheim, 1882; Chodat, 1902;  Wasser and Tsarenko, 2000; Wynne and Hallan, 2015; Wynne and Guiry, 2016 |  |
|  |  | *Scenedesmus acuminatus* (Lagerheim) Chodat |  |  |
|  |  | *Acutodesmus acuminatus* (Lagerheim) P.M.Tsarenko |  |  |
|  |  | *Tetradesmus* *acuminatus*(Lagerheim) M. J. Wynne |  |  |
| 14.2 | *T*. *lagerheimii* var. *biseriatus* (Reinhard) Taşkin & Alp | *Scenedesmus acuminatus* var. *biseriatus* Reinhard | Reinhard, 1904; Taşkın, 2019 |  |
| 14.3 | *T*. *lagerheimii* var. *tetradesmoides* | *Scenedesmus acuminatus* var. *tetradesmoides* G.M.Smith | Smith, 1916; Korshikov, 1953; Taşkın, 2019 |  |
|  |  | *Scenedesmus acuminatus* f. *tetradesmoides* (G.M.Smith) Korshikov |  |  |
| 15 | *T*. *lanceae* **sp. nov.** H. S. Cho & J. M. Lee |  | This study |  |
| 16.1 | *T*. *major* (Korshikov) Fott & Komárek |  | Fott and Komárek, 1974 |  |
| 16.2 | *T*. *mjaor* f. *lunatus* (Korshikov) Fott & Komárek | *Tetradesmus lunatus* Korshikov | Korshikov, 1953; Fott and Komárek, 1974 |  |
| 16.3 | *T*. *major* f. *petkoffi* (Printz) Fott & Komárek | *Tetradesmus petkoffi* Printz | Printz, 1915; Chodat, 1926; Fott and Komárek, 1974 |  |
|  |  | *Scenedesmus petkoffi* (Printz) Chodat |  |  |
| 17 | *T*. *nygaardii* (Huber-Pestalozzi) M.J.Wynne | *Scenedesmus nygaardii* Huber-Pestalozzi | Huber-Pestalozzi, 1936; Hegewald et al., 2013; Wynne and Hallan, 2015 |  |
|  |  | *Acutodesmus nygaardii* (Huber-Pestalozzi) E. Hegewald, C. Bock & Krienitz |  |  |
| 18.1 | *T*. *obliquus* (Turpin) M.J.Wynne | *Achnanthes obliqua* Turpin | Turpin, 1828; Meyen, 1829; Kützing, 1834; Hortobágyi, 1941; Hegewald and Hanagata, 2000;  Wynne and Hallan, 2015 |  |
|  |  | *Scenedesmus obliquus* (Turpin) Kützing |  |  |
|  |  | *Acutodesmus obliquus* (Turpin) E. Hegewald & N. Hanagata |  |  |
|  |  | *Scenedesmus acutus* Meyen |  |  |
|  |  | *Scenedesmus bijugatus* Kützing |  |  |
|  |  | *Scenedesmus acutus* f. *alternans* Hortobágyi |  |  |
| 18.2 | *T*. *obliquus* var. *alternans* (Krhistyuk) Taşkin & Alp | *Scenedesmus obliquus* var. *alternans* Khristyuk | Khristjuk, 1926; Taşkın, 2019 |  |
|  | *T*. *obliquus* var. *dactylococcoides* (Chodat) M.J.Wynne | *Scenedesmus dactylococcoides* Chodat | Chodat, 1926; Hegewald et al., 2013; Wynne and Hallan, 2015 |  |
|  |  | *Acutodesmus obliquus* var. *dactylococcoides* (Chodat) E. Hegewald, C. Bock & Krienitz |  |  |
| 18.6 | *T*. *obliquus* var. *flexuosus* (Lemmermann) Taşkin & Alp | *Scenedesmus bijugatus* var. *flexuosus* Lemmermann | Lemmermann, 1898; Collins, 1909; Taşkın, 2019 |  |
|  |  | *Scenedesmus bijugus* var. *flexuosus* (Lemmermann) Collins |  |  |
| 18.3 | *T*. *obliquus* f. *rectilineare* **f. nov.** H. S. Cho & J. M. Lee |  | This study |  |
| 18.4 | *T*. *obliquus* var. *spiraformis* **var. nov.** H. S. Cho & J. M. Lee |  | This study |  |
| 19 | *T*. *reginae* (T.Holtmann) M.J.Wynne | *Scenedesmus* (*Tetradesmus*) *wisconsinensis* var. *reginae* T. Holtmann | Holtmann, 1994; Hegewald and Hanagata, 2000; Tsarenko et al., 2005; Wynne and Hallan, 2015 |  |
|  |  | *Scenedesmus reginae* (T. Holtmann) E. Hegewald & N. Hanagata |  |  |
|  |  | *Acutodesmus reginae* (T. Holtmann) P. M. Tsarenko & E. Hegewald |  |  |
| 20 | *T*. *smithii* Prescott |  | Prescott, 1944 |  |
| 21.1 | *T*. *wisconsinensis* G. M. Smith | *Scenedesmus wisconsinensis* (G. M. Smith) Chodat | Smith, 1913; Chodat, 1913; Tsarenko and Petlevanny, 2001 |  |
|  |  | *Acutodesmus wisconsinensis* (G. M. Smith) P. M. Tsarenko |  |  |
| 21.2 | *T*. *wisconsinensis* f. *sibiricus* (Printz) Fott & Komárek | *Tetradesmus sibiricus* Printz | Printz, 1916; Fott and Komárek, 1974 |  |
| 21.3 | *T*. *wisconsinensis* f. *ostenfeldii* (Printz) Fott & Komárek | *Victoriella ostenfeldii* Wołoszyńska | Wołoszyńska, 1914; West, 1915; Fott and Komárek, 1974 |  |
|  |  | *Tetradesmus ostenfeldii* (Wołoszyńska) G. S. West |  |  |
