# Supplementary material for: Taxonomic reinvestigation of the genus Tetradesmus (Scenedesmaceae; Sphaeropleales) based on morphological characteristics and chloroplast genomes
Source: Front Plant Sci. 2024 Feb 14;15:1303175. doi: 10.3389/fpls.2024.1303175 (PMC10899504; doi:10.3389/fpls.2024.1303175)
Supplement: Supplementary file 2 [file DataSheet_1.pdf]

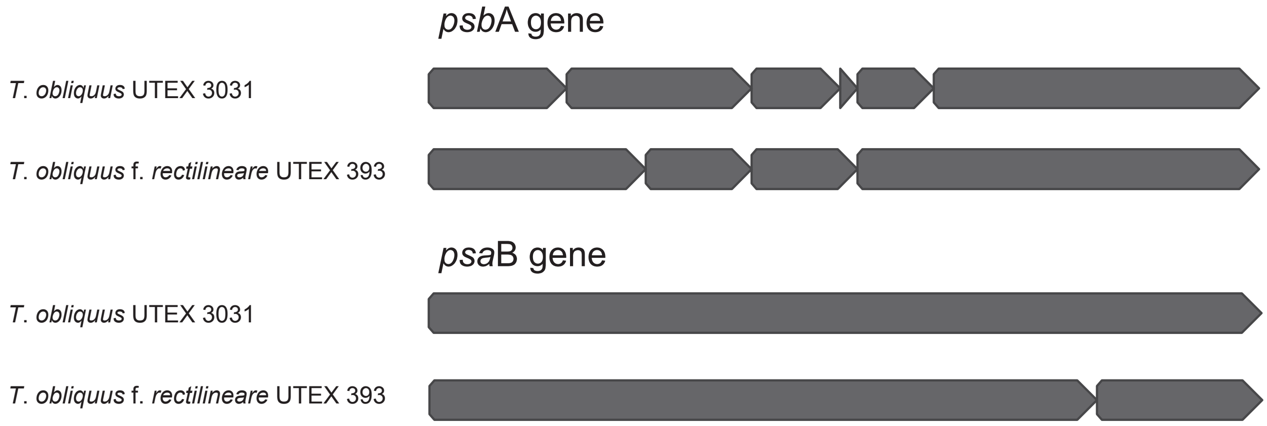

Supplementary Figure 1. Exon structures of *psb*A and *psa*B in *T. obliquus* strains (UTEX 393 and UTEX 3031).

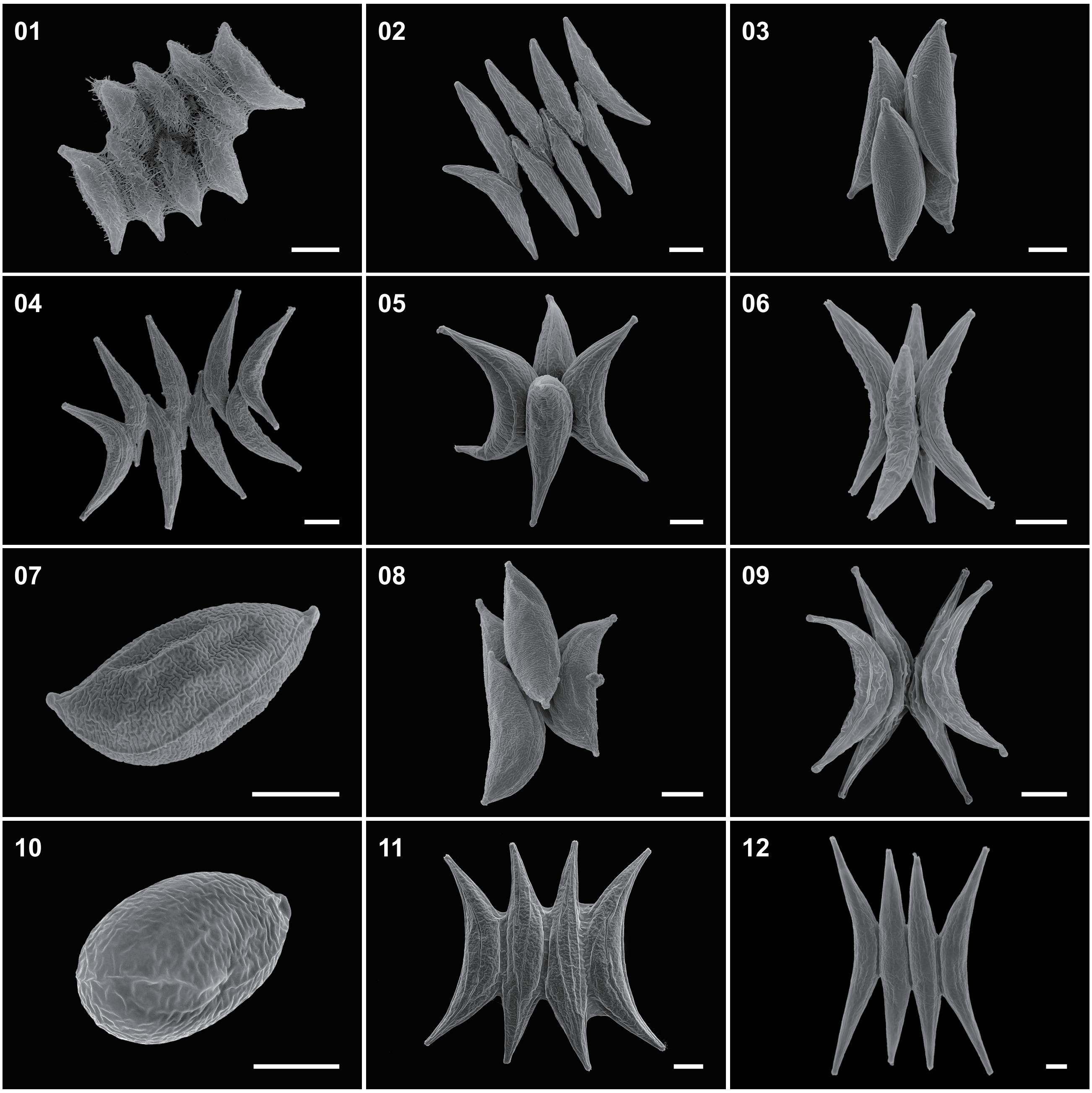

Supplementary Figure 2. Scanning electron microscopic images of *Tetradesmus* strains (scale bars: 3 µm). (A) *T. obliquus* UTEX 3031; (B) *T. obliquus* f. *rectilineare* UTEX 393; (C) *T. obliquus* var. *spiraformis* SAG 22.81; (D) *T. distendus* FBCC-A1020; (E) *T. major* f. *lunatus* FBCC-A1035; (F) *T. reginae* CCAP 276/66; (G) *T. bajacalifornicus* SAG 3.99; (H) *T. arenicola* SAG 2564; (I) *T.* cf. *lagerheimii* SAG 38.81; (J) *T. dissociatus* f. *oviformis* SAG 5.95; (K) *T. dimorphus* FBCC-A330; (L) *T. lancea* FBCC-A708.

(A)

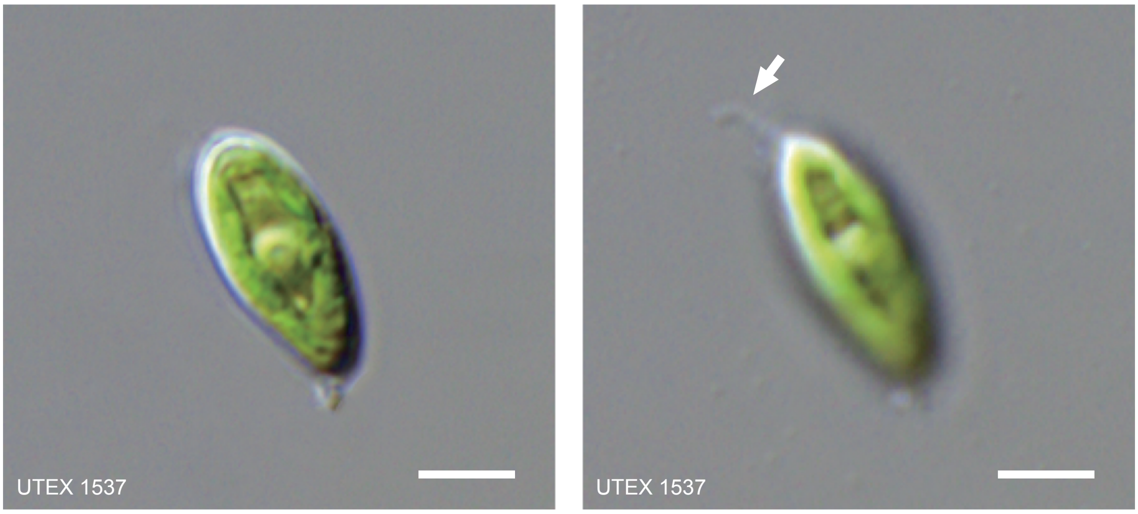

(B)

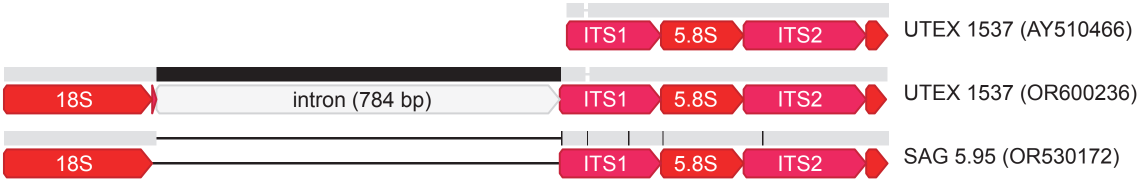

Supplementary Figure 3. *Tetradesmus dissociatus* UTEX 1537. (A) Light microscopic images of *T. dissociatus* UTEX 1537. The bridge-like structure (arrow) of the cell is indicated by an arrowhead (scale bars = 5 μm). (B) Multiple sequence alignment of the rRNA regions in UTEX 1537 (AY510466, Lewis and Flechtner, 2004; OR600236 from this study) and SAG 5.95 (OR530172).

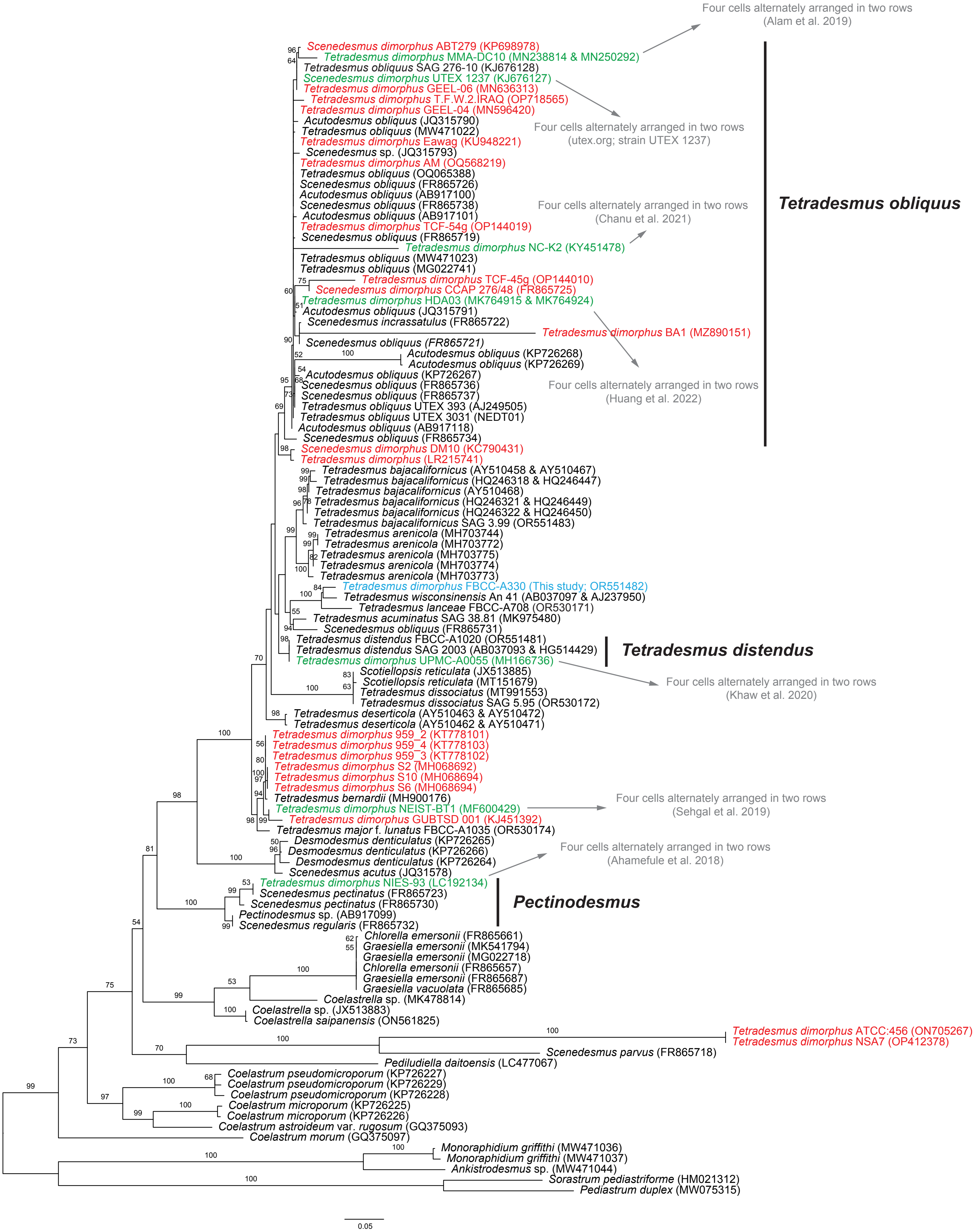

Supplementary Figure 4. The maximum likelihood tree of *Tetradesmus dimorphus* and related taxa constructed using ribosomal RNA (18S-ITS1-5.8S-ITS2) regions (only >50% bootstrap supporting values are shown). The sequences of *T. dimorphus* are highlighted by three colors as follows: red = sequence data only; green = available both sequence and morphological data; blue = *T. dimorphus* from this study.

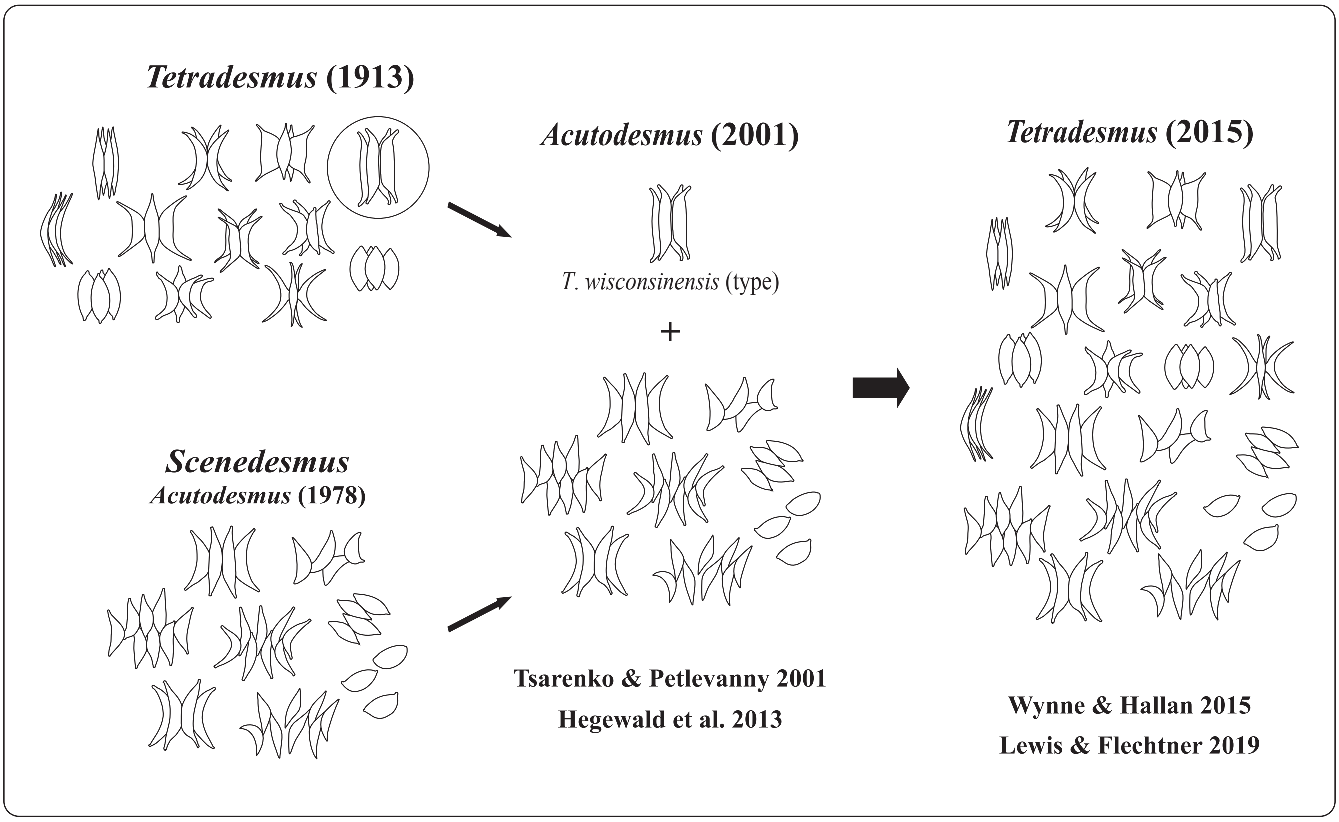

Supplementary Figure 5. Taxonomic history of the genus *Tetradesmus*.

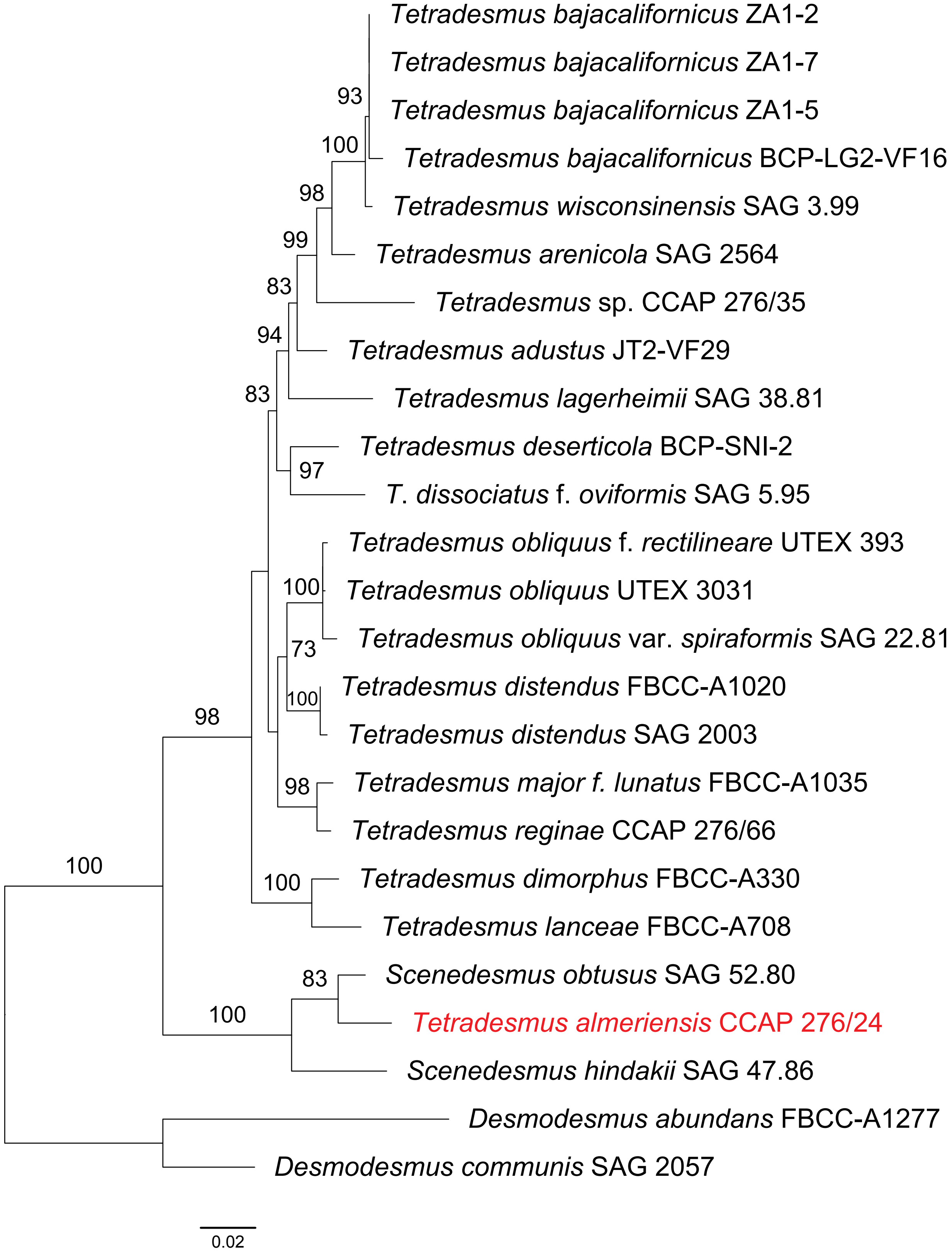

Supplementary Figure 6. The maximum likelihood tree of *Tetradesmus* (*Scenedesmus*) *almeriensis* (ITS, MF977406; *rbc*L, MG257492) and related taxa constructed using the ITS (ITS1-5.8S-ITS2) region, *rbc*L, and *tuf*A sequences (only >60% bootstrap supporting values are shown). Outgroup taxa are *Desmodesmus abundans* (ITS, OP103755; *rbc*L and *tuf*A from NC_066651) and *Desmodesmus communis* (*rbc*L, HG514364; *tuf*A, HG514391).

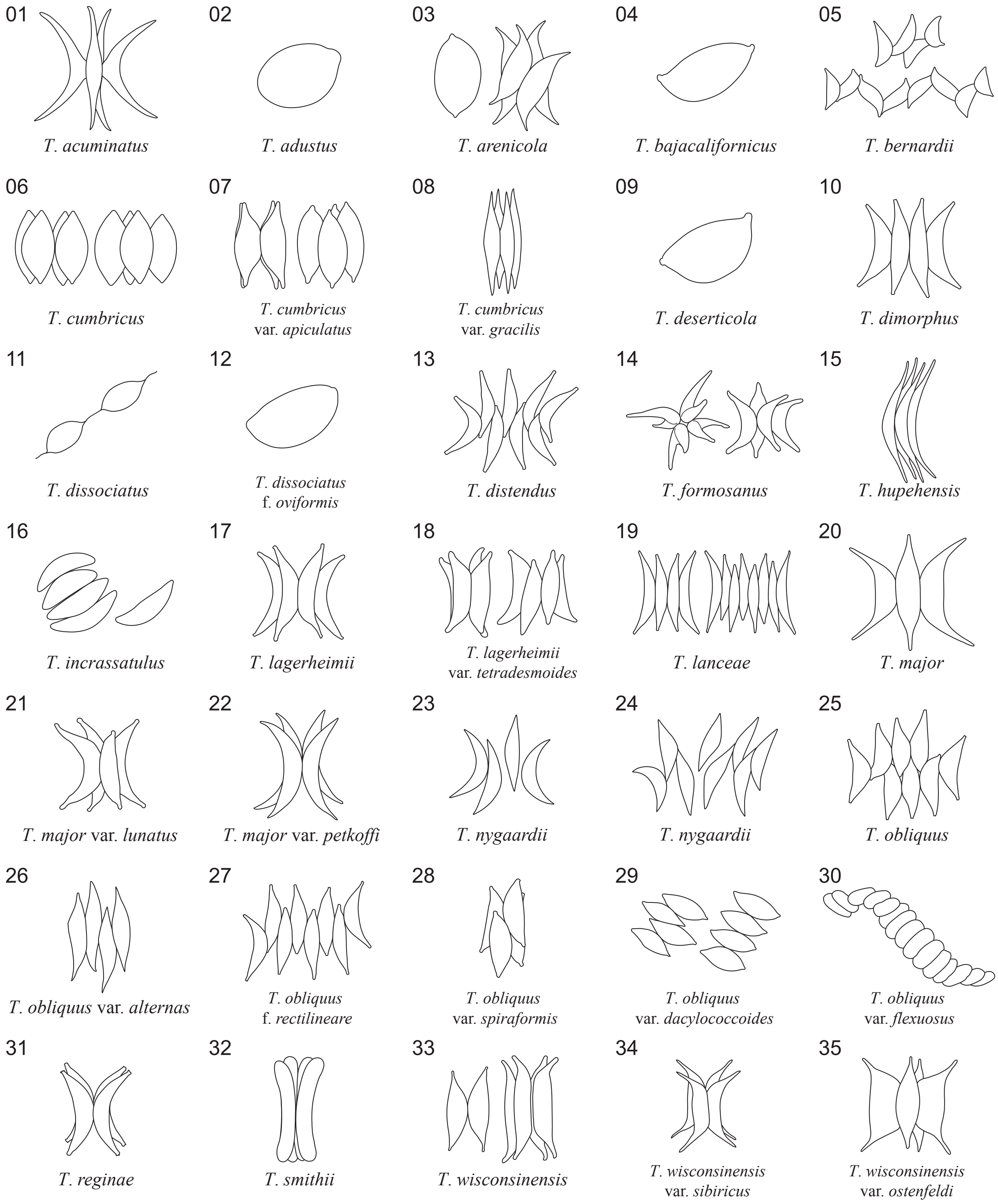

Supplementary Figure 7. Illustrations of *Tetradesmus* taxa based on original descriptions (Supplementary Table 1) and morphological observations of culture strains (this study).
